# Antibacterial and wound healing effects of PEG-coated ciprofloxacin-loaded ZIF-8 nanozymes against ciprofloxacin-resistant *Pseudomonas aeruginosa* taken from burn wounds

**DOI:** 10.3389/fphar.2025.1556335

**Published:** 2025-09-11

**Authors:** Mohadeseh Pahlevani, Masoumeh Beig, Seyed Mahmoud Barzi, Milad Sadeghzadeh, Morvarid Shafiei, Mohsen Chiani, Aria Sohrabi, Mohammad Sholeh, Shaghayegh Nasr

**Affiliations:** ^1^ Department of Microbial Biotechnology, University of Science and Culture, Faculty of Modern Biological Sciences and Technologies, Tehran, Iran; ^2^ Department of Bacteriology, Pasteur Institute of Iran, Tehran, Iran; ^3^ Microbiology Research Center (MRC), Pasteur Institute of Iran, Tehran, Iran; ^4^ Department of Biotechnology, Iranian Research Organization for Science and Technology (IROST), Tehran, Iran; ^5^ Department of Immunology, School of Public Health, Tehran University of Medical Sciences, Tehran, Iran; ^6^ Reproductive Immunology Research Center, Avicenna Research Institute, ACECR, Tehran, Iran; ^7^ Department of Nanobiotechnology, New Technology Research Group, Pasteur Institute of Iran, Tehran, Iran; ^8^ Department of Epidemiology and Biostatics, Research Centre for Emerging and Reemerging Infectious Diseases, Pasteur Institute of Iran, Tehran, Iran; ^9^ Microorganisms Bank, Iranian Biological Resource Center (IBRC), ACECR, Tehran, Iran

**Keywords:** *Pseudomonas aeruginosa*, zeolitic imidazolate framework-8 (ZIF-8), PEG-coated nanozymes, CIP-loaded nanozymes, burn wounds

## Abstract

**Background:**

Antimicrobial-resistant (AMR) *Pseudomonas aeruginosa* (*P. aeruginosa*) poses a significant challenge in burn wound infections due to its biofilm formation and resistance mechanisms, particularly against ciprofloxacin (CIP). Innovative therapies are urgently needed to improve treatment outcomes for burn patients. This study aimed to develop and evaluate Polyethylene glycol (PEG)-Coated CIP-Loaded zeolitic imidazolate framework-8 (ZIF-8) nanozymes (PEG-ZIF-8-CIP) to enhance antimicrobial efficacy against CIP-resistant *P. aeruginosa* (CRP) and promote wound healing.

**Methods:**

Clinical isolates of CRP were collected from burn patients and confirmed via polymerase chain reaction for the *oprL* gene. ZIF-8 nanozymes were synthesized, loaded with CIP, and coated with polyethylene glycol to form PEG-ZIF-8-CIP. These nanozymes were characterized using field emission scanning electron microscopy, Fourier-transform infrared spectroscopy, dynamic light scattering, and zeta potential measurements. Their antimicrobial efficacy, biofilm eradication capability, CIP release, and superoxide dismutase-like activity were assessed; Cytotoxicity Assay and wound healing effects were evaluated in a murine burn model infected with CRP. Statistical analyses were performed using ANOVA with Tukey correction in GraphPad Prism (v10.2.1), considering p-values < 0.05 as statistically significant.

**Results:**

Among 60 *P. aeruginosa* isolates, 40 were confirmed as ciprofloxacin-resistant (CRP) and carried the *oprL* gene. PEG-ZIF-8-CIP nanozymes achieved high drug entrapment efficiency (75%) and strong stability (zeta potential: –31.7 mV), with uniform spherical morphology (∼600 nm). Drug release followed a biphasic pattern—50% released in 6 h, ∼90% by 72 h. The nanozymes showed potent antimicrobial and antioxidant activity, with low MBECs and rapid absorbance reduction. Cytotoxicity was lowest for PEG-ZIF-8-CIP, especially at 24–48 h. *In vivo*, PEG-ZIF-8-CIP accelerated burn wound healing, reduced inflammation, promoted fibroblast growth and collagen deposition, and achieved the highest bacterial clearance (up to 84%).

**Conclusion:**

PEG-ZIF-8-CIP nanozymes effectively treated ciprofloxacin-resistant *P. aeruginosa* in burn-wound models by combining strong antimicrobial and anti-biofilm activity with improved wound healing. Encapsulation in ZIF-8 boosted antibiotic potency, while PEGylation enhanced stability, reduced toxicity, and enabled sustained drug release—highlighting their strong potential for combating antimicrobial-resistant wound infections.

## Graphical Abstract

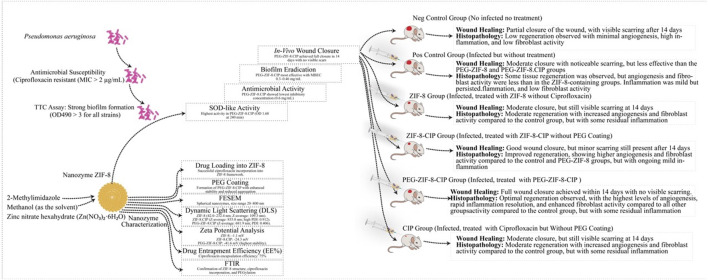

## 1 Introduction

Burn wounds are highly susceptible to multidrug-resistant (MDR) pathogens, with *Pseudomonas aeruginosa* (*P. aeruginosa*) being a leading cause of hospital-acquired infections, especially in immunocompromised and burn patients (Beig et al., 2021). The pathogen’s remarkable ability to form biofilms and deploy diverse resistance mechanisms, such as efflux pump overexpression, reduced membrane permeability, and biofilm-mediated protection, significantly limits the efficacy of conventional antibiotics like ciprofloxacin (CIP). Ciprofloxacin-resistant *P. aeruginosa* (CRP) poses a particularly grave challenge in burn wound infections, as it further narrows available therapeutic options (Chinemerem Nwobodo et al., 2022; Beig and Arabestani, 2019). These issues underscore an urgent need for innovative antimicrobial strategies to overcome resistance while promoting effective wound healing. Metal-organic frameworks (MOFs), particularly zeolite imidazolate frameworks (ZIFs) such as ZIF-8, have emerged as promising platforms for advanced antibacterial therapies. ZIF-8, a crystalline material composed of zinc ions and 2-methylimidazole linkers, exhibits high porosity, thermal stability, and pH-responsive drug delivery capabilities, making it ideal for sustained antibiotic release in infection environments (Shariati et al., 2022). ZIF-8 demonstrates intrinsic antibacterial properties through reactive oxygen species (ROS) generation and zinc ion release, which can disrupt bacterial membranes and reduce efflux pump activity, enhancing the uptake and efficacy of antibiotics like CIP (Costa et al., 2023; Yan et al., 2022). Additionally, ZIF-8 has been shown to disrupt biofilms, a critical feature for combating *P. aeruginosa* infections (Shariati et al., 2022; Costa et al., 2023; Yan et al., 2022). Polyethylene glycol (PEG) has been incorporated into ZIF-8 formulations to further improve its applicability in wound healing. PEG is a biocompatible and hydrophilic polymer that enhances stability, reduces cytotoxicity, and minimizes microbial adhesion of ZIF-8, making it a valuable component for biomedical applications (Costa et al., 2023). PEG coating also contributes to wound healing by promoting a hydrated environment conducive to tissue regeneration (Gallis et al., 2019; Wang et al., 2022b; Rahmanian et al., 2024). By combining the properties of ZIF-8 with PEG, the resultant PEG-coated ZIF-8 nanozymes exhibit superior antibacterial and wound-healing capabilities. This study takes this approach further by developing ciprofloxacin-loaded PEG-coated ZIF-8 nanozymes (PEG-ZIF-8-CIP) as a dual-action therapeutic agent. This formulation leverages the intrinsic antibacterial effects of ZIF-8, the biocompatibility and wound-healing support of PEG, and the potent antibacterial activity of CIP. The PEG-ZIF-8-CIP nanozymes are specifically designed to target CRP strains in burn wound infections, offering a solution that disrupts biofilms, reduces bacterial viability, and accelerates wound recovery (Rahmanian et al., 2024; Bagchi et al., 2019; Ahmed et al., 2020). Innovative approaches combining ZIF-8 with other nanozymes, such as glucose oxidase or gold nanoparticles, have further demonstrated enhanced antibacterial effects through increased ROS generation and biofilm disruption, paving the way for their use in complex clinical scenarios (Al Sharabati et al., 2022; Li et al., 2024). These advancements highlight the versatility of ZIF-8-based nanozymes in addressing the dual challenges of antibiotic-resistant infections and effective wound healing. The present study optimizes PEG-ZIF-8-CIP nanozymes for enhanced therapeutic efficacy against CRP in burn wounds. Key parameters, such as particle size, zeta potential, entrapment efficiency, and stability, are evaluated to ensure optimal formulation properties. The research also investigates their antimicrobial activity, biofilm-disrupting potential, and wound-healing effects *in vitro* and *in vivo*. By addressing the limitations of current treatments, this study aims to provide a novel and effective therapeutic strategy for improving clinical outcomes in burn patients, particularly those affected by pathogens like *P. aeruginosa* (Shariati et al., 2022; Costa et al., 2023; Yan et al., 2022; Zhang et al., 2023; Wang et al., 2022a; Yao et al., 2020; Gallis et al., 2019; Wang et al., 2022b; Rahmanian et al., 2024; Bagchi et al., 2019; Ahmed et al., 2020; Al Sharabati et al., 2022; Li et al., 2024; Wan et al., 2021).

## 2 Materials and methods

### 2.1 Clinical isolation and characterization of *P. aeruginosa*

#### 2.1.1 Isolation and biochemical identification

Samples were collected from patients hospitalized at Shahid Motahari Hospital in Tehran, Iran, from January 2024 to August 2024. Eighty burn wound exudate samples were collected from patients with burns using a swabbing technique and transferred to the bacteriology department of the Pasteur Institute of Iran for clinical examination. Then, samples were cultured on blood agar plates for 24 h at 37 °C to isolate pure colonies. Isolates were initially diagnosed as *P. aeruginosa* following biochemical tests. Finally, the isolates were kept in brain heart infusion media containing 20% glycerol and stored at −70 °C (Beig et al., 2021).

#### 2.1.2 DNA extraction

The total DNA of *P. aeruginosa* isolates was extracted using the boiling method. In this procedure, three to five colonies from an overnight bacterial culture were suspended in 500 µL of sterile distilled water, boiled for 30 min, and centrifuged at 14,000 × g for 5 min to remove cell debris. The extracted DNA was then stored at −20 °C. The DNA’s quantity and quality were assessed using a NanoDrop ND-1000 spectrophotometer (Biocompare, San Francisco, United States) and gel electrophoresis (Wang et al., 2022c).

#### 2.1.3 Molecular detection of the *oprL* gene

Polymerase chain reaction (PCR) amplification was performed to detect the *oprL* gene as a housekeeping gene that targets a 504 bp region. Primers were designed using BLAST analysis on the NCBI platform and synthesized by Pishgam Biotech Co. The reaction mixture (25 µL total) included 12.5 µL Taq DNA Polymerase Master Mix RED (AMPLIQON, Denmark), 1 µL of each primer, 1 µL template DNA, and 9.5 µL nuclease-free water. PCR reactions were conducted in sterile PCR tubes on ice and amplified using an Eppendorf thermal cycler under the following conditions: initial denaturation at 94 °C for 4 min, followed by 30 cycles of denaturation (94 °C for 30 s), annealing (57 °C for 45 s), and extension (72 °C for 1 min). A final extension step at 72 °C for 5 min completed the reaction. Products were analyzed on 1.5% agarose gels, stained with ethidium bromide, and visualized under UV light using a Bio-Rad gel documentation system (Wong et al., 2015).

#### 2.1.4 Antimicrobial susceptibility testing using minimum inhibitory concentration

Antimicrobial susceptibility testing for CIP in *P. aeruginosa* was performed using the minimum inhibitory concentration (MIC) method, following the Clinical and Laboratory Standards Institute (CLSI 2023) guidelines (Clinical and Laboratory, 2023). The MIC test was conducted using the standard microdilution method, with *P. aeruginosa* ATCC 27853 and *Escherichia coli* ATCC 25922 used as control strains. A bacterial suspension was prepared from a 24-h culture on Mueller-Hinton agar, adjusted to a McFarland standard of 0.5, and confirmed using a spectrophotometer at 625 nm. This suspension was diluted to achieve a final 1.6 × 10^8^ CFU/mL concentration. Serial dilutions of CIP were prepared in 96-well microtiter plates, with 10 µL of the bacterial suspension added to each well. Positive control wells contained the bacterial suspension without CIP, while negative control wells contained only Mueller-Hinton broth. Plates were incubated at 37 °C for 24 h, and the MIC was defined as the lowest concentration of CIP that inhibited visible bacterial growth. Strains were considered resistant to CIP if the MIC was ≥2 mg/L, according to CLSI guidelines for systemic therapy (Beig et al., 2020a).

#### 2.1.5 Biofilm formation assay using 2,3,5-triphenyl tetrazolium chloride method

In this study, we assessed the biofilm-forming ability of *P. aeruginosa* strains using the 2,3,5-Triphenyl Tetrazolium Chloride (TTC) method (Sabaeifard et al., 2014). Bacterial cultures were initially inoculated into 5 mL of LB broth and incubated overnight at 37 °C with shaking at 200 rpm. The overnight cultures were then diluted to an optical density (OD) of 0.1 at 600 nm to standardize the inoculum. Next, 100 µL of the bacterial suspension was added to each well of a 96-well microtiter plate, with negative control wells containing sterile LB broth and positive control wells containing a known biofilm-forming strain. The plate was incubated at 37 °C for 24 h to allow biofilm formation. After incubation, the planktonic cells were carefully aspirated, and the biofilm in each well was washed with 100 µL of sterile phosphate-buffered saline (PBS). To stain the biofilms, 100 µL of 0.5% TTC solution was added to each well, and the plate was incubated at 37 °C for 2–3 h, enabling the reduction of TTC to red-colored formazan by metabolically active cells within the biofilm. After incubation, the TTC solution was removed, and the formazan was solubilized by adding 100 µL of isopropanol to each well, followed by gentle shaking for 10 min. The absorbance at 490 nm was measured using a microplate reader to quantify the metabolic activity of the biofilm. The background absorbance from control wells was subtracted, and the resulting data were used to compare biofilm formation between different *P. aeruginosa* strains. Statistical analysis was performed to assess significant differences in biofilm production across strains. This method provides a reliable measurement of biofilm metabolic activity, though additional techniques, such as field emission scanning electron microscopy (FESEM), could be used for further structural analysis of the biofilms.

### 2.2 Synthesis of nanozymes

#### 2.2.1 Formulation of ZIF-8 and PEG-ZIF-8-CIP nanozymes

ZIF-8 nanozymes were synthesized via a solvothermal method. Zinc nitrate hexahydrate (Zn(NO_3_)_2_·6H_2_O) and 2-methylimidazole were dissolved in methanol at a 1:8 M ratio and stirred at room temperature for 24 h. The resulting ZIF-8 crystals were centrifuged at 10,000 rpm for 10 min, washed with methanol, and vacuum-dried at 60 °C.

#### 2.2.2 Ciprofloxacin loading and PEGylation

PEG-coated ZIF-8-CIP nanozymes were prepared by encapsulating CIP (1 mg/mL) in ZIF-8 with continuous stirring for 6 h, followed by PEG coating (mass ratio 1:1) for 12 h. The PEG-coated nanozymes were centrifuged, washed, and dried at 60 °C (Zhang et al., 2018).

### 2.3 *In vitro* characterization and bioactivity

#### 2.3.1 Physicochemical characterization of nanozymes

The ZIF-8 and PEG-ZIF-8-CIP nanozymes were characterized for particle size, size distribution, and zeta potential using a Zetasizer Nano ZS3600 with a 633 nm He-Ne laser. Morphological analysis was performed using FESEM. Fourier-transform infrared spectroscopy (FTIR) was conducted using a Bruker FT-IR VERTEX 70, while dynamic light scattering (DLS) and zeta potential measurements were performed using a HORIBA Scientific SZ-100. The stability of the particles was evaluated over 1 month by monitoring size, polydispersity index (PDI), zeta potential, and drug entrapment efficiency (EE%) (Jongert et al., 2024).

#### 2.3.2 Long-term colloidal stability assessment

Zeta potential measurements were conducted over 180 days at room temperature to assess the long-term colloidal stability of the prepared formulations. The analysis was performed using DLS system equipped with a zeta potential analyzer. Samples, including ZIF-8, ZIF-8-CIP, PEG-ZIF-8-CIP, and free CIP, were dispersed in distilled water at a consistent concentration and stored in sealed vials under ambient conditions. Zeta potential values were recorded at specific time points (0, 30, 60, 90, 120, 150, and 180 days) to monitor stability.

#### 2.3.3 Drug entrapment efficiency

To assess drug entrapment, 15 mg of ZIF-8 was incubated in 30 mL of ethanolic CIP solution (0.5 mg/mL) with stirring at room temperature in a light-protected shaker. Supernatants were collected at 12, 24, and 48 h to evaluate EE% using UV-vis spectroscopy at 273 nm (Costa et al., 2023). The EE% was calculated as follows:

$$\text{Entrapment efficiency }\left(\text{EE}\%\right)=\frac{\text{Weight}\;\text{of}\;\text{CIP}\;\text{in}\;\text{ZIF}\;‐\;8}{\text{weight}\;\text{of}\;\text{CIP}\;\text{fed}\;\text{initially}}\times 100$$

#### 2.3.4 Ciprofloxacin release

CIP release from PEG-ZIF-8 was evaluated in phosphate-buffered saline (PBS, pH 7.4) at 37 °C under sink conditions. For each experiment, 0.15 g of CIP-loaded PEG-ZIF-8 was dispersed in 45 mL of pre-warmed PBS and magnetically stirred at 200 rpm. At 0.5, 1, 2, 3, 4, 24, 48, and 72 h, 5 mL aliquots of the release medium were removed and immediately replaced with an equal volume of fresh, 37 °C PBS to maintain constant volume and sink conditions. The amount of CIP released in each sample was determined by UV–Vis spectrophotometry at 276 nm using a calibration curve. To elucidate the underlying kinetics, release data were fitted (GraphPad Prism v10.2.1) to zero-order, first-order, Higuchi, and Korsmeyer–Peppas models (see Table 1).

**TABLE 1:** CIP release kinetics from PEG-ZIF-8.

Model	k	*R* ^2^	n
Zero Order	1.8	−0.16	—
First Order	0.25	0.94	—
Higuchi	15.07	0.68	—
Korsmeyer-Peppas	34.73	0.96	0.26

#### 2.3.5 Superoxide dismutase-like activity

The superoxide dismutase (SOD) activity of ZIF-8 and ZIF-8-CIP nanozymes was evaluated using the nitroblue tetrazolium (NBT) reduction assay (Li et al., 2022). For the assay, a solution of NBT (100 μg/mL in DMSO) was prepared and mixed with the nanozyme suspensions at a concentration of 1 mg/mL. The reaction mixture was incubated, and color changes were observed to indicate superoxide radical scavenging. Absorbance was measured at 529 nm at 30-min intervals using a spectrophotometer. The intensity of the color, directly proportional to the amount of superoxide radicals present, was used to determine the SOD-like activity of the nanozymes.

### 2.4 *In vitro* antimicrobial efficacy

#### 2.4.1 Disc diffusion assay

The disc diffusion method evaluated the antimicrobial efficacy of ZIF-8, ZIF-8-CIP, and PEG-ZIF-8-CIP nanozymes. Bacterial suspensions were standardized to the McFarland 0.5 standard and cultured on Mueller-Hinton agar. Blank discs loaded with various nanozyme concentrations (10, 20, 30, 40, and 50 mg/mL) were placed on the agar and incubated at 37 °C for 24 h. Inhibition zones were measured to determine the antimicrobial efficacy of the nanozymes.

#### 2.4.2 Minimum biofilm eradication concentration

The minimum biofilm eradication concentration (MBEC) assay evaluated the efficacy of CIP-loaded nanozymes in eradicating biofilms formed by CRP (Verma and Maheshwari, 2017). The MBEC was determined using a 96-well microtiter plate method, following the protocol established by Moskowitz et al. Bacterial suspensions (108 CFU/mL) were prepared by culturing *P. aeruginosa* isolates in tryptic soy broth (TSB) medium (Merck, United States) at 37 °C for 24 h. A 20 µL aliquot of the suspension was added to sterile 96-well plates containing 180 µL of TSB medium supplemented with 1% glucose to promote biofilm formation. The plates were incubated at 37 °C for 1, 3, or 5 days to allow biofilm development. After incubation, non-adherent cells were removed by washing the wells thrice with 200 µL of sterile saline. Biofilms were then treated with 100 µL of nanozyme solutions from 10 to 50 m concentrations mg/mL. The plates were incubated for 24 h at 37 °C, and the wells were rinsed thrice with sterile saline. The wells were then stained using a TTC method. The plates were read with an automated ELISA reader (Titertek, R Multiscan) at 470 nm wavelength.

#### 2.4.3 Cytotoxicity assay

For the 3-(4,5-Dimethylthiazol-2-yl)-2,5-diphenyltetrazolium bromide (MTT) assay, human foreskin fibroblasts (HFF; Pasteur Institute of Iran) were seeded in 96-well plates at 10 × 10^3^ cells per well in 100 µL of complete DMEM/F12 medium and incubated overnight for cell adherence. The following day, cells were treated with ZIF-8, ZIF-8-CIP, PEG-ZIF-8-CIP nanozymes, or CIP, each resuspended in complete DMEM/F12 medium at concentrations ranging from 10 to 100,000 ng/mL. Cells treated with complete DMEM/F12 medium alone served as the control group. After 24, 48, or 72 h of incubation, the media were carefully removed, and 100 µL of MTT solution (1 mg/mL in PBS; Sigma-Aldrich) was added to each well. Plates were incubated for 3 h at 37 °C. Following incubation, the MTT solution was aspirated, and 100 µL of dimethyl sulfoxide (DMSO; Sigma-Aldrich) was added to solubilize the formazan crystals. Absorbance was measured at 570 nm using a microplate reader (BioTek).

### 2.5 *In vivo* evaluation

#### 2.5.1 Animal ethics and grouping

Female BALB/c mice (6–9 weeks, 18–20 g) were obtained from the Pasteur Institute and allowed to acclimate for 1 week before experimentation. The mice were randomly assigned to one of six treatment groups, each consisting of five animals: (I) Wounded Negative Control: Wounded mice treated with sterile distilled water, (II) Positive Control: Wounded, infected, and untreated mice; (III) ZIF-8 Group: Wounded, infected mice treated with ZIF-8 nanozyme, (IV) ZIF-8-CIP treated group: Wounded, infected mice treated with CIP-loaded ZIF-8 nanozyme, (V) PEG-ZIF-8-CIP Group: Wounded, infected mice treated with PEGylated CIP-loaded ZIF-8 nanozyme (PEG-ZIF-8-CIP), and (VI) CIP Treated Group: Wounded, infected mice treated with CIP.

#### 2.5.2 Wound formation and infection model

To induce anesthesia, mice were administered an intraperitoneal injection of ketamine (60 mg/kg) and xylazine (10 mg/kg) (Ahmadi-Noorbakhsh et al., 2022). The dorsal fur (between vertebrae L2-L6) was shaved using shaving cream, and the wound sites were sterilized. A 5 mm incision was made with a biopsy punch, targeting the epidermis and superficial dermis without affecting the deeper musculature. The wounds were infected with 0.5 McFarland suspension of a CRP suspension (1.5 × 10^8^ CFU/mL), except for the negative control group, which did not receive bacterial inoculation. Following infection, the wounds were treated daily with 20 µL of ZIF-8, ZIF-8-CIP, or PEG-ZIF-8-CIP nanozymes for 14 days. After infection wound creation, a small amount of medical-grade adhesive (3M Vetbond) was applied directly to the wound site to secure the treatment area. Subsequently, the wound and surrounding dorsal skin were carefully covered with zinc oxide adhesive tape extending from the posterior to the anterior part of the mouse. This ensured complete coverage and helped maintain the stability and retention of the administered nanozyme formulations throughout the treatment period. Following the treatment period, the mice were euthanized with an overdose of anesthesia. Each group was kept in their post-operation cage on a 12-h light/dark cycle with access to food, tap water, and libitum and received human care.

#### 2.5.3 Wound bacterial count

Wound bacterial count was performed using CFU Assay. At 24 h after wound induction, mice in all treatment groups except the positive control received a single topical dose of one of the following: ZIF-8, ZIF-8-CIP, PEG-ZIF-8-CIP Nanozymes, and CIP. Wound swabs were collected post-treatment on days 1, 3, 7, and 10 to monitor bacterial clearance.

For each time point, exudate was harvested by gently swabbing the wound bed with a sterile cotton swab pre-moistened in 2 mL of sterile distilled water. The swab was immediately transferred to a tube containing 3 mL of sterile physiological saline and vortexed to release adherent bacteria. A 100 μL suspension aliquot was plated onto Muller agar (Merck, Germany) and incubated at 37 °C for 24 h. Colonies were counted and expressed as CFU per wound to quantify the antibacterial efficacy of each formulation.

#### 2.5.4 Wound healing evaluation

Photographs of the wounds on each mouse were taken to evaluate the wound healing progression, and their diameters were recorded on days 1, 3, 7, and 14 after the injury. Precise measurements were obtained using digital calipers to maintain consistency across all observations. The extent of wound contraction, which reflects the healing process, was calculated as the percentage reduction in wound size using the following formula:

$$\text{Wound contraction }\left(\%\right)=\left(A0\;–\text{ AT}\right)$$

A0 represents the initial wound area measured immediately after the injury, while AT corresponds to the wound area measured on subsequent treatment days.

#### 2.5.5 Histopathological analysis

Histopathological analysis followed the methodology outlined in Abed et al. (2024). A detailed scoring system (Khalaf et al., 2019) was employed to evaluate and compare histopathological changes across various experimental groups. This structured approach ensured precise assessment and offered comprehensive insights into the changes observed under different experimental conditions. The histopathological analysis assessed the therapeutic effects of ZIF-8, ZIF-8-CIP, and PEG-ZIF-8-CIP nanozymes on drug-resistant *P. aeruginosa* infections. On the 14th day post-operation, the healed area on the dermis was removed and kept in a 10% formalin solution for further pathology investigations. The healed areas on the dermis were removed and preserved in formalin for further investigation. Hematoxylin and eosin (H&E) staining was performed to evaluate the inflammatory response (neutrophil infiltration), fibroblast activity, epithelial regeneration, and neovascularization as indicators of wound healing. These parameters were assessed by counting neutrophils, fibroblasts, epithelial layers, and blood vessels. All animal procedures were conducted according to ethical guidelines and were approved by the Ethical Committee of the Pasteur Institute of Iran (IR.PII.REC.1398.025).

### 2.6 Statistical analyses

Statistical analyses were conducted using one-way and two-way ANOVA tests, with the Tukey *post hoc* test applied for correction [GraphPad Prism, version 10.2.1 (339)]. A p-value of less than 0.05 was considered statistically significant.

## 3 Results

### 3.1 Clinical isolation and molecular confirmation of *P. aeruginosa*

#### 3.1.1 Isolation and biochemical identification

Among 80 isolates, 60 strains of *P. aeruginosa* were isolated and identified by biochemical methods.

#### 3.1.2 Molecular detection of the *oprL* gene

Molecular analysis revealed the presence of the *oprL* gene in 60 isolates, confirmed by PCR from a distinct band of 504 bp (Supplementary Figure 1A).

#### 3.1.3 Minimum inhibitory concentration of ciprofloxacin

MIC testing identified 60 *P. aeruginosa* isolates resistant to CIP with MICs greater than 2 μg/mL (Supplementary Figure 1B).

#### 3.1.4 Biofilm-forming assay by TTC assay

Biofilm formation was evaluated by measuring the absorbance at 490 nm (OD490) after staining the biofilms with TTC. Based on the OD490 values, biofilm formation intensity was classified into three categories: weak biofilm formation (OD490 < 0.5), moderate biofilm formation (OD490 between 0.5 and 1), and strong biofilm formation (OD490 > 1). In the experiments conducted, the OD490 values for the tested *P. aeruginosa* strains were consistently higher than 3, indicating a powerful biofilm-forming capacity across most strains. These results suggest that the strains evaluated in this study exhibited robust biofilm formation, with metabolic activity significantly surpassing the threshold for strong biofilm production.

### 3.2 *In vitro* characterization of nanozymes

#### 3.2.1 Field emission scanning electron microscopy analysis of ZIF-8 and ZIF-8-CIP

**FIGURE 1:**
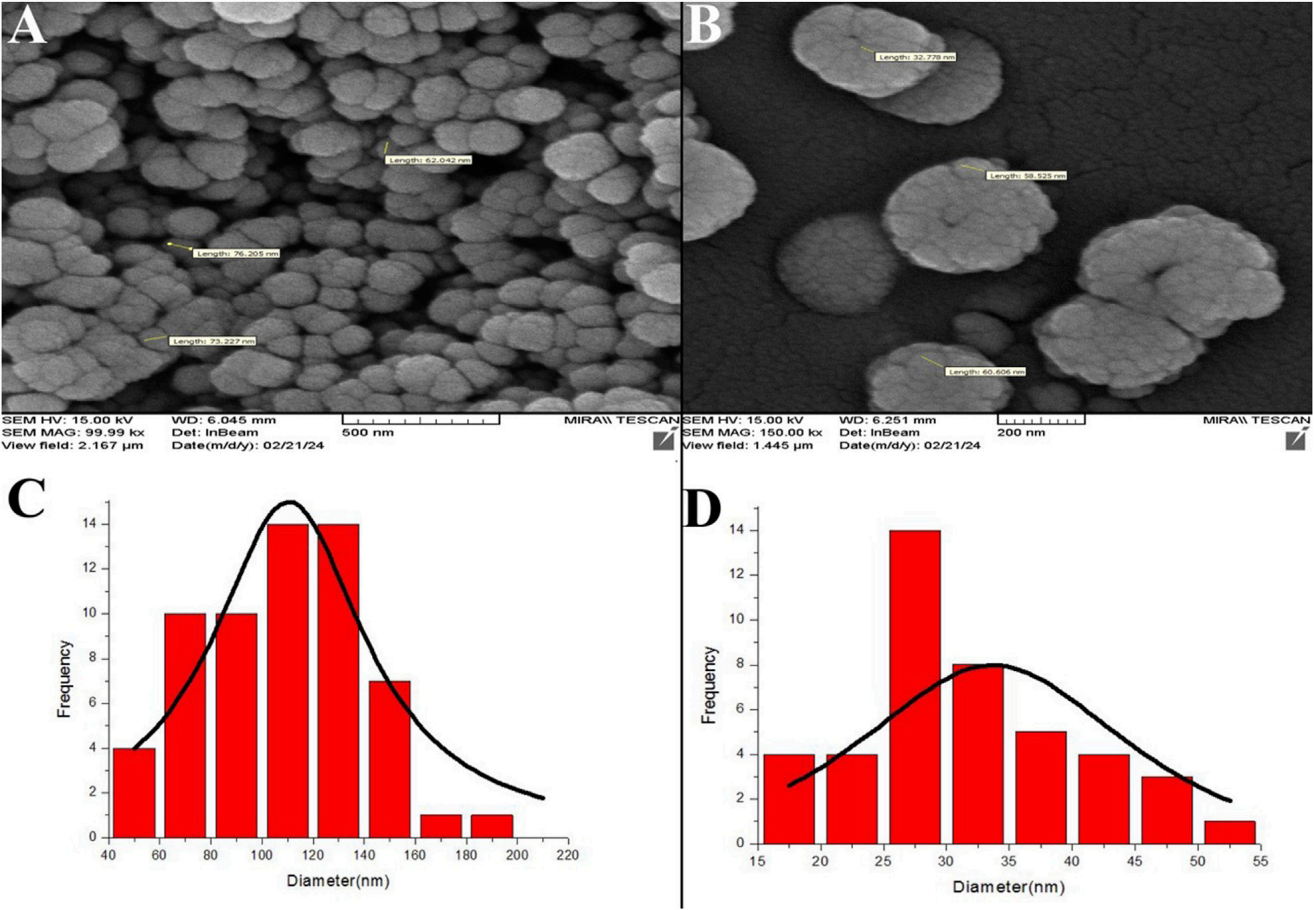
FESEM micrographs depicting ZIF-8 nanozymes **(A)** and ZIF-8-CIP nanozymes **(B)**. The particle diameters of the nanozymes were analyzed and calculated using ImageJ software. The left side of the FESEM microscopic image of ZIF-8-CIP nanozymes, and the right side of the FESEM microscopic image of ZIF-8. nanozymes. Size distribution histograms of ZIF-8 **(C)** and ZIF-8-CIP nanozymes **(D)**.

FESEM analysis revealed ZIF-8 and ZIF-8. CIP nanozymes are spherical, with sizes ranging from 20 to 200 nm (Figure 1).

The micrographs in Figures 1A,B illustrate the morphology of ZIF-8 and ZIF-8-CIP nanozymes, respectively. ZIF-8 nanozymes displayed a tightly packed arrangement with a slightly broader size range, while ZIF-8-CIP nanozymes exhibited larger and more loosely packed particles. Incorporating CIP in ZIF-8-CIP nanozymes appeared to influence particle size and morphology, contributing to minor variations in the structural arrangement. Size distribution histograms were generated using ImageJ software to quantify particle diameters. The histogram for ZIF-8 nanozymes (Figure 1C) displayed a broader particle size distribution. In contrast, the histogram for ZIF-8-CIP nanozymes (Figure 1D) showed a more concentrated size range, reflecting the effect of CIP loading on particle uniformity. These findings confirm the structural integrity and controlled size characteristics of ZIF-8-based nanozymes, with CIP incorporation inducing slight modifications in particle morphology.

#### 3.2.2 Dynamic light scattering analysis of hydrodynamic size, PDI, and stability

**FIGURE 2:**
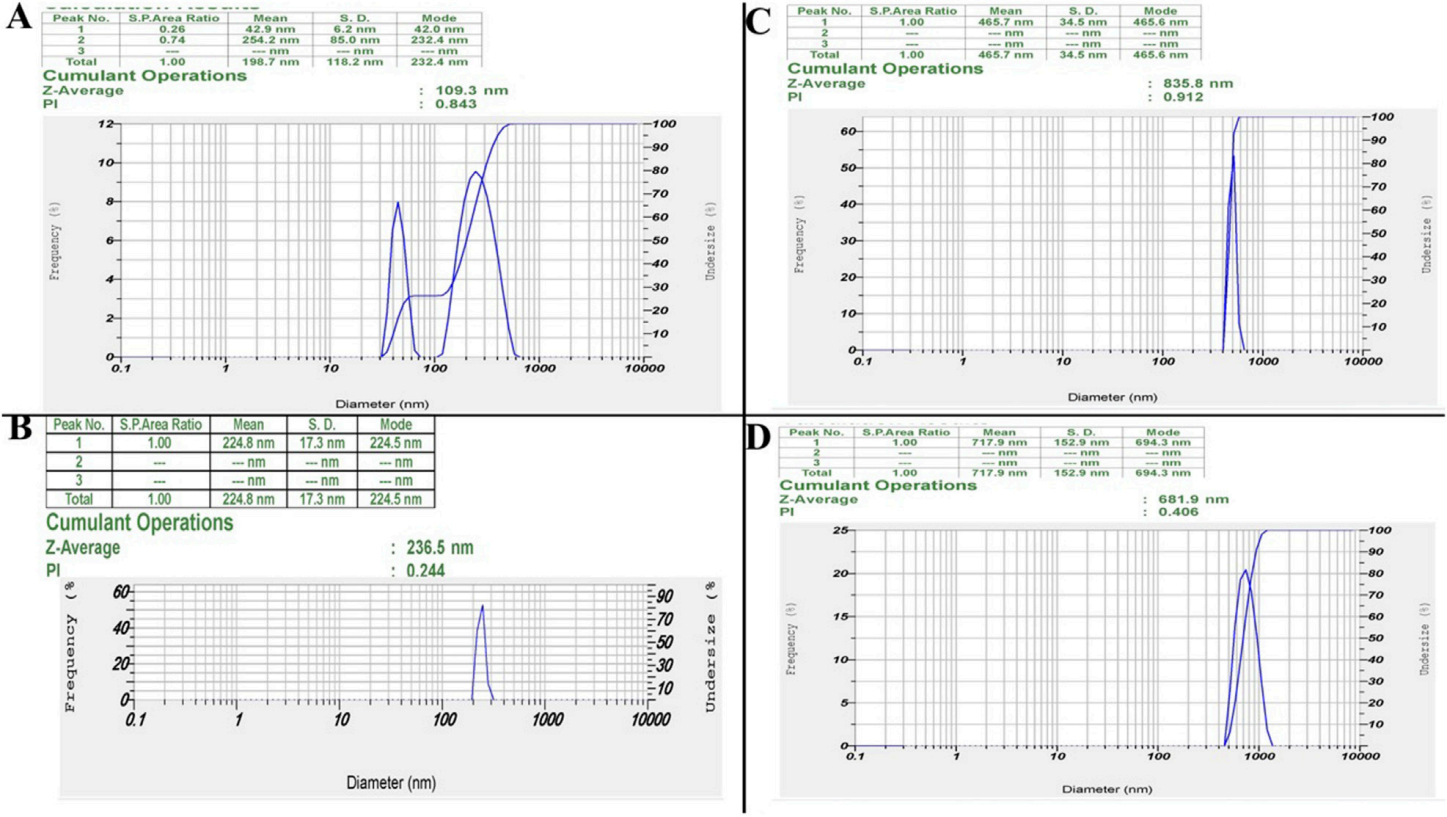
Dynamic light scattering (DLS) results. Particle Size Distributions of ZIF-8, ZIF-8-CIP, and PEG-ZIF-8-CIP Nanozymes. **(A)** ZIF-8 Nanozymes: DLS analysis revealed a bimodal size distribution with 42.0 and 232.4 nm peaks. This resulted in a Z-average size of 109.3 nm and a polydispersity index (PDI) of 0.843, indicating moderate polydispersity. **(B)** CIP: DLS analysis of CIP displayed a single-size distribution with a mean particle size of 224.8 nm and a PDI of 0.244, suggesting a relatively uniform particle size distribution. **(C)** ZIF-8-CIP Nanozymes: CIP-loaded ZIF-8 enzymes exhibited a single-size distribution with a Z-average size of 835.8 nm and a high PDI of 0.912, reflecting broader size distribution and increased heterogeneity compared to ZIF-8 alone. **(D)** PEG-ZIF-8-CIP Nanozymes: PEGylation of ZIF-8-CIP resulted in a Z-average size of 681.9 nm and a PDI of 0.406 post-stability testing, suggesting reduced heterogeneity and aggregation compared to pre-stability conditions.

DLS analysis revealed distinct particle size distributions and stability characteristics for ZIF-8, CIP, ZIF-8-CIP, and PEG-ZIF-8-CIP nanozymes (Figure 2). The ZIF-8 nanozymes (Figure 2A) exhibited a bimodal size distribution with peaks at 42.0 and 232.4 nm, resulting in a Z-average size of 109.3 nm and a PDI of 0.843, indicating moderate polydispersity. CIP (Figure 2B) displayed a single-size distribution with a mean particle size of 224.8 nm and a PDI of 0.244, reflecting a relatively uniform particle size distribution. For ZIF-8-CIP nanozymes (Figure 2C), the incorporation of CIP resulted in a larger particle size, with a Z-average size of 835.8 nm and a high PDI of 0.912, indicating a broader size distribution and increased heterogeneity compared to ZIF-8 alone. PEGylation of ZIF-8-CIP nanozymes (Figure 2D) significantly reduced the particle size post-stability testing, with a Z-average size of 681.9 nm and a decreased PDI of 0.406, suggesting reduced heterogeneity and aggregation. These results highlight the effects of CIP loading and PEGylation on particle size and stability with PEG-ZIF-8. After stability testing, CIP demonstrated improved stability and reduced heterogeneity.

#### 3.2.3 Zeta potential measurement

**FIGURE 3:**
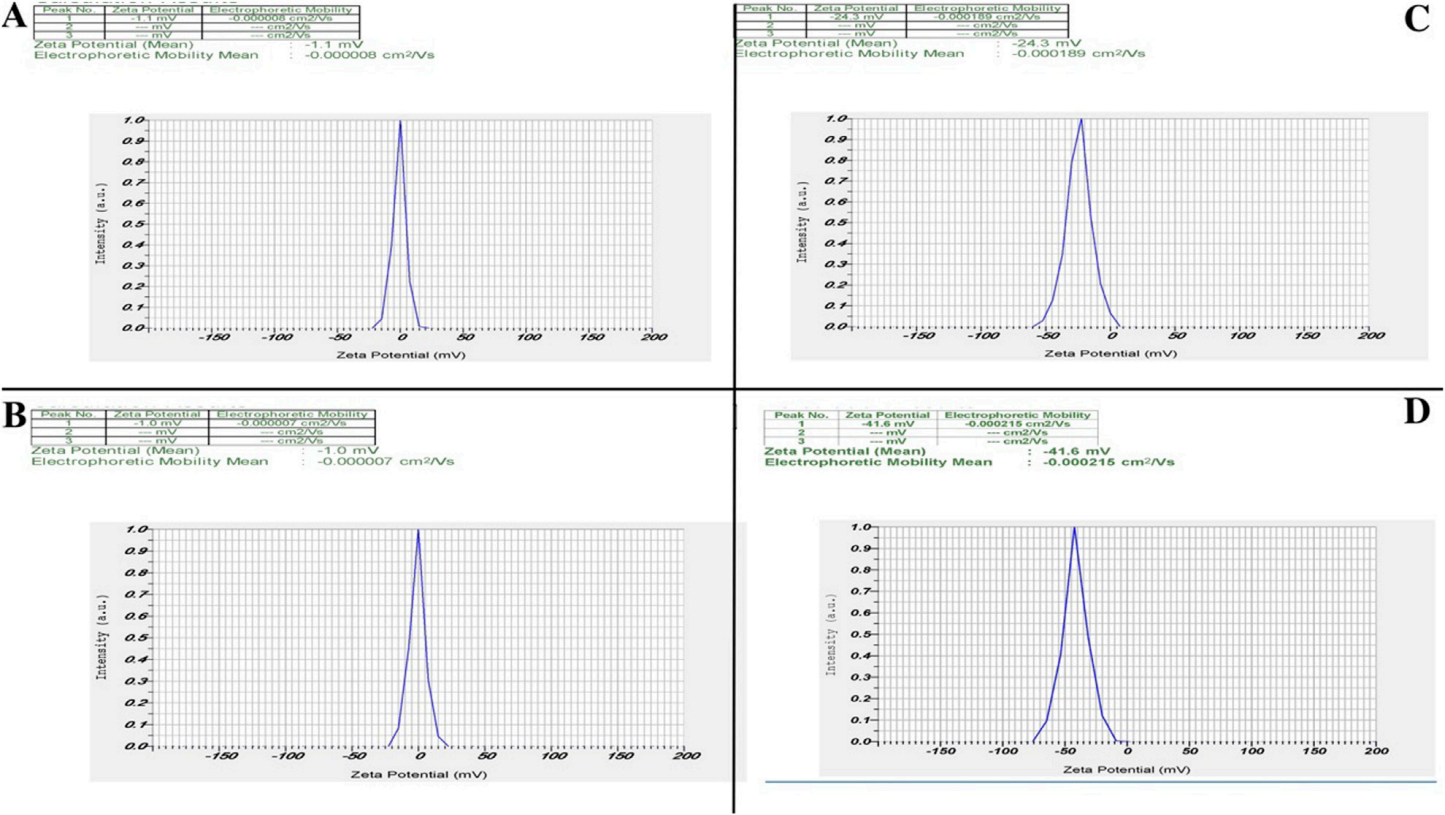
Zeta potential analysis of nanozymes. Zeta potential analysis revealed distinct differences among the nanozymes, highlighting their stability and surface charge characteristics. **(A)** ZIF-8 nanozymes exhibit a zeta potential of −1.1 mV, indicating a low surface charge and limited stability. **(B)** CIP alone shows a zeta potential of −1.0 mV, reflecting minimal surface charge. **(C)** ZIF-8-CIP nanozymes demonstrate an increased zeta potential of −24.3 mV, confirming successful CIP incorporation and improved stability. **(D)** PEG-coated ZIF-8-CIP nanozymes exhibit the highest zeta potential of −41.6 mV, indicating enhanced stability due to PEGylation, which provides steric protection and further increases surface charge.

Zeta potential measurements demonstrated a clear enhancement in surface charge—and thus colloidal stability across our nanozyme formulations (Figure 3). Unmodified ZIF-8 (Figure 3A) nanoparticles exhibited a near-neutral potential of −1.1 mV, reflecting minimal electrostatic repulsion and poor stability. Free CIP (Figure 3B) similarly showed −1.0 mV. CIP into the ZIF-8 (Figure 3C) framework shifted the surface charge to −24.3 mV, confirming successful drug loading and a substantial increase in electrostatic stabilization. PEGylation further improved stability: PEG-ZIF-8-CIP (Figure 3D) nanozymes reached −41.6 mV, indicating that steric shielding by PEG chains and higher net surface charge effectively resists aggregation.

#### 3.2.4 Long-term colloidal stability

**FIGURE 4:**
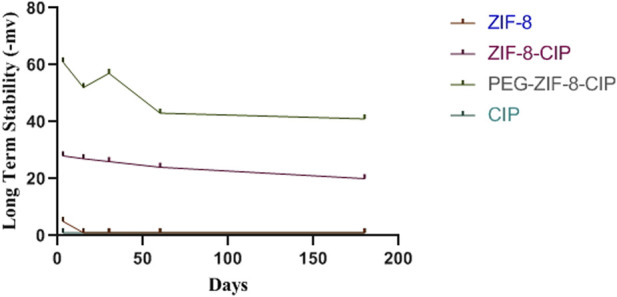
Long-term colloidal stability of nanozymes. The zeta potential of empty ZIF 8 (red), ciprofloxacin-loaded ZIF 8 (ZIF 8 CIP; purple), PEG-ZIF 8-CIP (dark green), and free CIP (light green) were measured at 0, 7, 30, 90, and 180 days of storage. Measurements were performed in aqueous suspension at room temperature. ZIF 8 started at −5 mV, fluctuated slightly during the first month, and stabilized at around −1 mV at day 180. PEG-ZIF-8-CIP had an initial −61 mV that decreased to −41 mV after 6 months.

To evaluate long-term stability, we monitored each formulation’s zeta potential over 180 days (Figure 4). Unmodified ZIF-8 began at −5 mV, drifted minimally in the first month, then settled near −1 mV by day 180. Incorporating CIP into ZIF-8 shifted the starting potential to −28 mV, and it gradually rose to −20 mV, reflecting partial charge neutralization while preserving colloidal integrity. PEGylated ZIF-8–CIP exhibited the greatest electrostatic resilience: its potential declined from −61 mV to just −41 mV over 6 months, underscoring PEG’s steric stabilization. By contrast, free CIP remained at approximately zero throughout, confirming its propensity to aggregate in aqueous media.

#### 3.2.5 FTIR spectra of ZIF-8, CIP, ZIF-8-CIP, and PEG-ZIF-8-CIP

**FIGURE 5:**
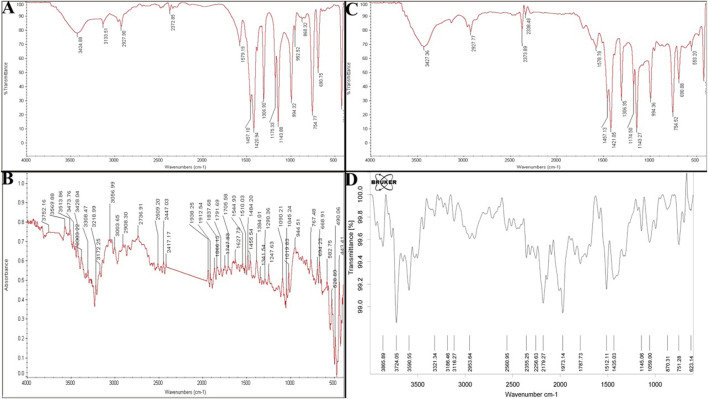
**(A)** FTIR Spectrum of CIP (control), ZIF-8, ZIF-8-CIP, and PEG-ZIF-8-CIP. FTIR spectrum of ZIF-8 nanozyme. Reveals its structural integrity with characteristic peaks for O-H, N-H, and imidazole-related vibrations, confirming the stability of the ZIF-8 framework. **(B)** FTIR spectrum of CIP highlights its functional groups, including O-H, N-H, C=O, and C-F stretching vibrations, validating its chemical structure. **(C)** FTIR spectrum of ZIF-8-CIP nanozyme shows successful CIP incorporation, with peaks for C=O, C=N, and hydrogen bonding interactions. **(D)** FTIR spectrum of PEG-ZIF-8-CIP nanozyme confirms PEGylation and the retention of functional groups from ZIF-8 and CIP, ensuring stability and functionality.

The FTIR analysis provided comprehensive insights into the structural and chemical properties of ZIF-8-based nanozymes and their interactions with CIP and PEG modifications. For ZIF-8 nanozymes, characteristic peaks confirmed the structural integrity of the framework, including O-H stretching at 3,424 cm^-1^, N-H and C-H stretching vibrations of the imidazole ligand at 3,133 cm^-1^, and C=N and C=C stretching in the imidazole ring at 1,579 cm^-1^. Additional peaks at 1,175, 1,143, 1,306, and 1,420 cm^-1^ corresponded to C-N and C-C vibrations in the imidazole structure, while a distinct peak at 559 cm^-1^ was attributed to zeolitic vibrations, confirming the framework’s stability (Figure 5A).

The analysis of CIP highlighted the presence of its key functional groups, including hydroxyl (O-H) at 3,752 cm^-1^, amine (N-H) stretching between 3,513 and 3,569 cm^-1^, carbonyl (C=O) stretching at 1705 cm^-1^, and aromatic C=C and C=N bonds. C-F stretching vibrations at 1,019 cm^-1^ and bending vibrations for methyl and methylene groups confirmed CIP’s chemical structure (Figure 5B). For ZIF-8-CIP, nanozymes and FTIR spectra revealed notable shifts and additional peaks, indicating the successful incorporation of CIP into the ZIF-8 framework. A peak between 1700 and 1720 cm^-1^ corresponded to the carbonyl (C=O) absorption from the carboxylic acid groups of CIP, while the pyridine core of the fluoroquinolone ring was characterized by C=N stretching at 1,600 cm^-1^. Peaks between 1,400 and 1,450 cm^-1^ reflected bending vibrations of methyl and methylene groups, and C-F stretching vibrations appeared between 1,000 and 1,100 cm^-1^. A peak indicated hydrogen bonding and N-H interactions at 3,427 cm^-1^, and alkyl stretching vibrations at 2,850–2,950 cm^-1^ further confirmed the formation of the ZIF-8-CIP complex (Figure 5C).

The FTIR spectra of PEG-ZIF-8-CIP nanozymes displayed distinct peaks associated with both CIP and the PEGylated ZIF-8 framework, including N-H and O-H stretching vibrations between 3,100 and 3,300 cm^-1^ and C=O vibrations from the carboxylic acid groups of CIP in the range of 1,700–1,900 cm^-1^. These findings confirmed the successful PEGylation of ZIF-8-CIP nanozymes and the retention of key functional groups from ZIF-8 and CIP, resulting in a functional and stable PEG-ZIF-8-CIP nanozyme (Figure 5D).

### 3.3 Drug loading and release behavior

#### 3.3.1 Entrapment efficiency of CIP in ZIF-8

The entrapment efficiency (EE%) of CIP in ZIF-8 nanozymes was evaluated using UV-Vis spectrophotometry. After 72 h, the encapsulated amount of CIP was determined to be 0.01 g, corresponding to an absorbance of 0.042. The high entrapment efficiency, estimated at approximately 75%, highlights the effectiveness of the nanozyme structure in encapsulating the drug. These results demonstrate the successful incorporation of CIP into the ZIF-8 framework and its potential for prolonged and controlled drug release.

**FIGURE 6:**
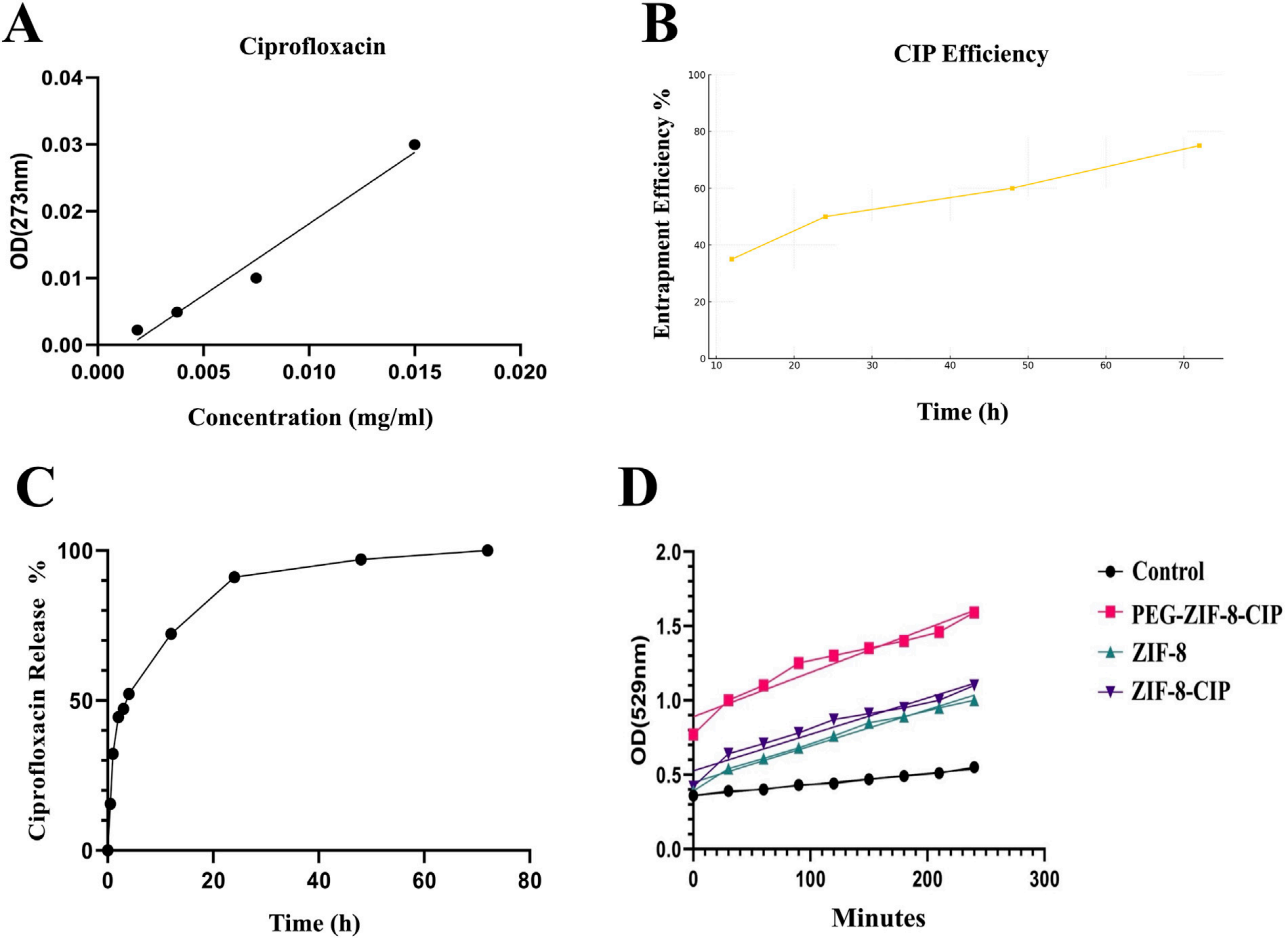
**(A)** Calibration curve for ciprofloxacin quantification and **(B)** time-dependent entrapment efficiency of ZIF-8-CIP. **(C)** *In vitro* Kinetic Analysis of CIP Release from PEG-ZIF-8 Nanoparticles. Cumulative release of CIP from PEG-ZIF-8 nanoparticles in PBS (pH 7.4) at 37 °C over 72 h. The profile shows an initial burst (≈50% release in the first 6 h), followed by sustained release reaching ≈90% by 72 h. **(D)** SOD-Like Activity of Nanozymes Over Time. The graph depicts the SOD-like activity of control (black circles), ZIF-8 (green triangles), ZIF-8-CIP (purple inverted triangles), and PEG-ZIF-8-CIP (pink squares) based on OD at 529 nm over 240 min. PEG-ZIF-8-CIP (pink squares) demonstrated the highest catalytic activity, followed by ZIF-8-CIP (purple inverted triangles) and ZIF-8 (green triangles). The control group (black circles) exhibited minimal activity throughout the experiment. The progressive increase in OD values for PEG-ZIF-8-CIP reflects the enhanced catalytic efficiency resulting from PEGylation and CIP incorporation.

Using the calibration line in Figure 6A, the absorbance of the 72 h supernatant (0.042 at 273 nm) corresponds to a residual CIP concentration of 0.020 mg/mL, indicating that 75% of the initial drug was encapsulated within ZIF-8. Plotting entrapment efficiency versus loading time (Figure 6B) reveals a time-dependent uptake: efficiency rises sharply from 35% at 12 h to 50% at 24 h, then more gradually to 60% at 48 h and 75% at 72 h. This trend confirms that extended incubation enhances CIP incorporation into the MOF pores, achieving maximal loading by 72 h.

#### 3.3.2 *In vitro* ciprofloxacin release

*In vitro* CIP release from PEG-ZIF-8 nanoparticles was carried out in PBS (pH 7.4) at 37 °C over 72 h. As illustrated in Figure 6C, the release profile was distinctly biphasic: an initial burst, with approximately 50% of the payload released within the first 6 h (p < 0.05), attributed to the rapid diffusion of drug molecules loosely adsorbed on the nanoparticle surface; followed by a prolonged, controlled release phase that reached roughly 90% cumulative release by 72 h, reflecting gradual diffusion from the inner pores of the ZIF-8 framework modulated by the PEG shell.

Among these, the Korsmeyer–Peppas model exhibited the best correlation (*R* ^2^ = 0.96) with an exponent n = 0.26 indicative of Fickian diffusion (n < 0.45 for spherical matrices). The first-order model also provided a strong fit (*R* ^2^ = 0.94), suggesting concentration-dependent release, whereas the zero-order (*R* ^2^ = −0.16) and Higuchi (*R* ^2^ = 0.68) models failed to describe the observed behavior adequately.

#### 3.3.3 Superoxide dismutase-like catalytic activity

The SOD-like catalytic activity of ZIF-8 and ZIF-8-CIP nanozymes was evaluated using the NBT reduction assay, with absorbance measured at 529 nm over 240 min. As shown in Figure 6D significant differences in the SOD activity among the various samples, highlighting the impact of different modifications on enzyme performance. At the 120-min mark, the absorbance values for ZIF-8, ZIF-8 with CIP (ZIF-8-CIP), PEG-coated ZIF-8 with CIP (PEG-ZIF-8-CIP), and the control were 0.76, 0.87, 1.30, and 0.44, respectively, indicating variations in enzymatic activity under different conditions. ZIF-8 alone exhibited the lowest activity (0.76), likely due to structural limitations restricting access to the active sites. When CIP was added to ZIF-8, a slight increase in SOD activity was observed (0.87), potentially due to CIP’s role in enhancing electron transfer or reducing oxidative stress. Combining PEG with ZIF-8-CIP further improved enzymatic activity, reaching 1.30 at 120 min. This enhancement is likely due to the increased stability and better dispersion of the nanoparticles, which will enhance enzyme accessibility and reduce inhibitory effects. Notably, PEG-ZIF-8-CIP demonstrated the highest activity (1.30), suggesting that the synergistic interactions between PEG, ZIF-8, and CIP optimize the overall catalytic performance. Compared to the control group (0.44), all experimental samples showed a significant increase in SOD-like activity, confirming the effectiveness of ZIF-8 and the role of PEG coating and CIP loading in boosting enzymatic function.

Over the entire 240-min incubation period, ZIF-8-CIP reached a peak absorbance of 1.2, substantially higher than the 0.89 observed for ZIF-8, indicating a marked improvement in catalytic efficiency due to incorporating CIP. ZIF-8-CIP maintained the highest OD at 529 nm throughout the experiment, with values of 0.8 at 90 min and 1.2 at 240 min, reflecting its rapid and efficient superoxide radical scavenging capability. In contrast, ZIF-8 reached a maximum OD of 0.89 at 240 min and 0.63 at 90 min, demonstrating more moderate SOD activity. The control group, which exhibited minimal or no SOD-like activity, consistently showed the lowest OD values (0.29–0.40), further confirming the catalytic action of ZIF-8 and ZIF-8-CIP.

PEG-coated ZIF-8 with CIP (PEG-ZIF-8-CIP) demonstrated the highest overall activity, with an OD of 1.68 at 240 min and 1.35 at 90 min. This peak activity reflects the synergistic interactions between PEG, ZIF-8, and CIP, which enhance the system’s SOD-like function. PEG, ZIF-8, and CIP significantly enhance the SOD-like activity, making PEG-ZIF-8. CIP is the most effective system in this study.

### 3.4 *In vitro* antimicrobial efficacy

#### 3.4.1 Disc diffusion assay

**FIGURE 7:**
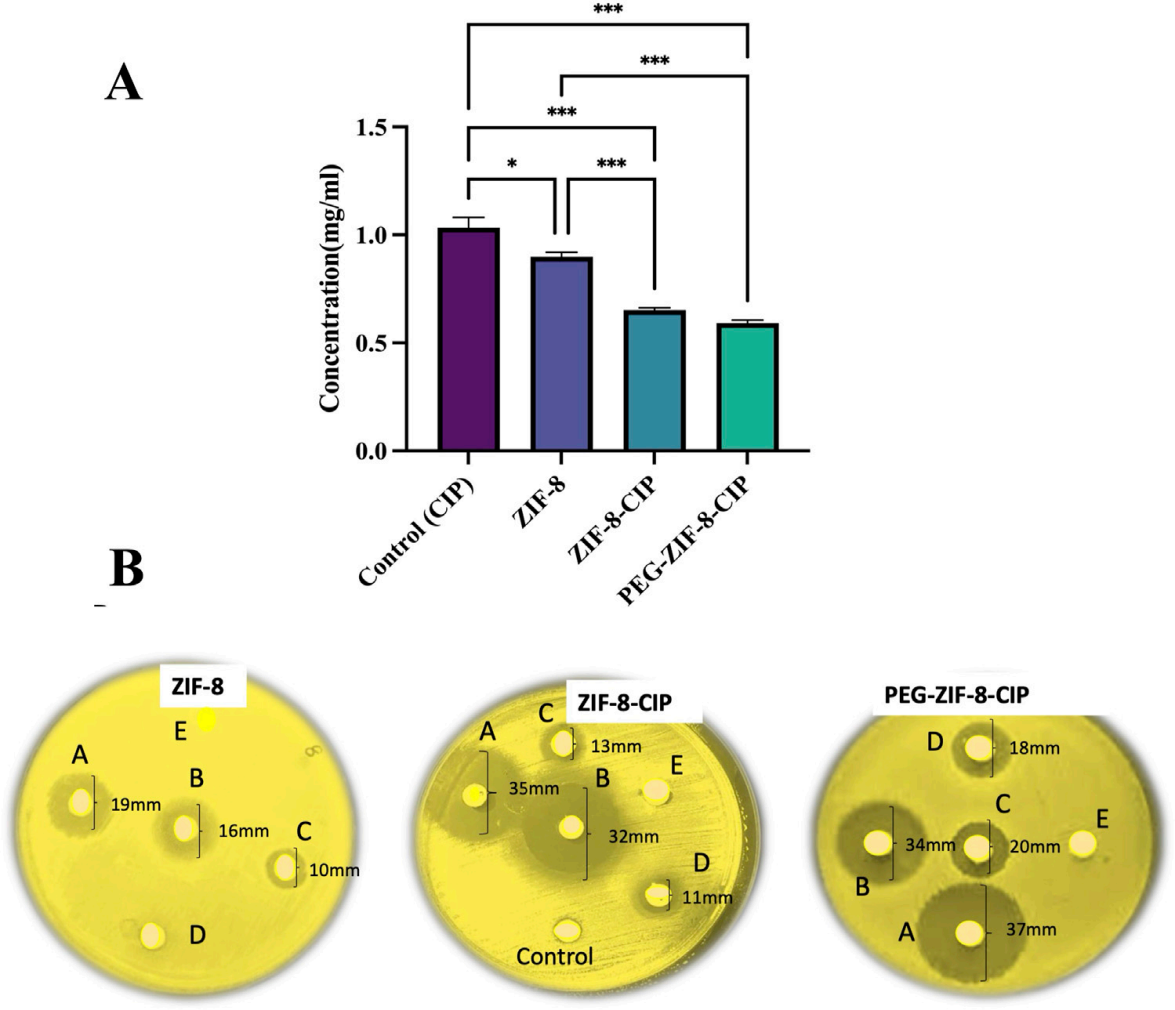
Disk Diffusion Assay of CIP (control), ZIF-8, ZIF-8-CIP, and PEG-ZIF-8-CIP **(A)** The disk diffusion assay evaluated the antimicrobial activity of CIP (control), ZIF-8, ZIF-8-CIP, and PEG-ZIF-8-CIP. The treatments are represented with distinct colors for clarity: CIP (control), ZIF-8, ZIF-8-CIP, and PEG-ZIF-8-CIP. The concentrations required for bacterial inhibition were compared across treatments. Statistically significant differences between groups are marked with asterisks (*p < 0.05, **p < 0.01, ***p < 0.001). Error bars indicate standard deviations **(B)** Disks **(A–E)** correspond to nanoparticle concentrations of 50 mg/mL **(A)**, 40 mg/mL **(B)**, 30 mg/mL **(C)**, 20 mg/mL **(D)**, and 10 mg/mL **(E)**. Left (ZIF-8 alone): The disk loaded with ZIF-8 alone produced a clear inhibition halo of 19 mm [**(A)**, 50 mg/mL], 16 mm [**(B)**, 40 mg/mL], and 10 mm [**(C)**, 30 mg/mL]. Disks **(D)** (20 mg/mL) and **(E)** (10 mg/mL) showed no halo formation. Middle (ZIF-8-CIP): Ciprofloxacin loading markedly enhanced efficacy, with zones of 35 mm [**(A)**, 50 mg/mL], 32 mm [**(B)**, 40 mg/mL], 13 mm [**(C)**, 30 mg/mL] and 11 mm [**(D)**, 20 mg/mL]. Neither the blank control disk nor disk **(E)** (10 mg/mL) produced any inhibition. Right (PEG-ZIF-8-CIP): PEGylation further improved antibiotic delivery, resulting in the largest halos of 37 mm [**(A)**, 50 mg/mL], 34 mm [**(B)**, 40 mg/mL], 20 mm [**(C)**, 30 mg/mL] and 18 mm [**(D)**, 20 mg/mL]. No inhibition was observed at 10 mg/mL **(E)**.

The antimicrobial efficacy of CIP (control), ZIF-8, ZIF-8-CIP, and PEG-ZIF-8-CIP was evaluated using a disk diffusion assay to determine the concentrations required for bacterial inhibition. The results showed that CIP alone required the highest concentration (1.1 mg/mL) to inhibit bacterial growth. ZIF-8 demonstrated an improved antimicrobial effect with a lower required concentration of 0.8 mg/mL. ZIF-8-CIP further enhanced efficacy, achieving bacterial inhibition at 0.7 mg/mL. Among all treatments, PEG-ZIF-8-CIP exhibited the greatest antimicrobial potency, requiring the lowest concentration (0.6 mg/mL) for inhibition, reflecting the synergistic effects of PEGylation and CIP loading on nanozyme performance (Figures 7A,B). Tukey’s multiple comparisons test revealed statistically significant differences among the treatments. CIP (control) showed significantly lower antimicrobial activity than all ZIF-8-based treatments. The comparison between CIP and ZIF-8 yielded a mean difference of 0.135 (*p* = 0.012), indicating a moderate improvement with ZIF-8. The effect was more pronounced for CIP versus ZIF-8-CIP, with a mean difference of 0.382 (*p* < 0.001), reflecting enhanced efficacy from CIP encapsulation. PEG-ZIF-8-CIP demonstrated tremendous improvement over CIP, with a mean difference of 0.442 (*p* < 0.001), showcasing the synergistic effects of PEGylation and CIP loading.

Comparisons among the ZIF-8-based treatments provided additional insights. ZIF-8-CIP significantly outperformed ZIF-8, with a mean difference of 0.247 (*p* < 0.001), indicating that CIP encapsulation greatly enhances antimicrobial efficacy. Similarly, PEG-ZIF-8-CIP showed superior activity to ZIF-8, with a mean difference of 0.307 (*p* < 0.001). However, the comparison between ZIF-8-CIP and PEG-ZIF-8-CIP revealed no statistically significant difference (mean difference: 0.060; *p* = 0.433), suggesting that while PEGylation provides significant benefits over ZIF-8 alone, its relative effect compared to ZIF-8-CIP is not substantial. These findings emphasize that PEG-ZIF-8-CIP delivers the most important improvement in antimicrobial activity compared to CIP and other ZIF-8 formulations. The statistical significance in most comparisons underscores the critical roles of CIP encapsulation and PEGylation in enhancing the therapeutic efficacy of ZIF-8-based nanozymes.

#### 3.4.2 Minimum biofilm eradication concentration

The anti-biofilm activity of ZIF-8, ZIF-8-CIP, and PEG-ZIF-8-CIP nanozymes was evaluated against *P. aeruginosa* biofilms at 1, 3, and 5 days of age, with MBEC values determined for each formulation. The results revealed that ZIF-8 alone exhibited increasing MBEC values as the biofilms matured, requiring 0.72 mg/mL for 1-day-old biofilms, 0.62 mg/mL for 3-day-old biofilms, and 0.76 mg/mL for 5-day-old biofilms, reflecting enhanced resistance with biofilm maturation. In contrast, ZIF-8-CIP nanozymes incorporating CIP demonstrated improved anti-biofilm efficacy. The MBEC values for ZIF-8-CIP were 0.46 mg/mL for 1-day-old biofilms, 0.62 mg/mL for 3 -3-day-old biofilms, and 0.64 mg/mL for 5 -5-day-old biofilms, indicating a more potent biofilm-eradicating effect compared to ZIF-8 alone. Among all the formulations tested, PEG-ZIF-8-CIP nanozymes exhibited the most robust anti-biofilm activity. This formulation showed MBEC values of 0.3 mg/mL for 1-day-old biofilms, 0.42 mg/mL for 3-day-old biofilms, and 0.46 mg/mL for 5-day-old biofilms. These results highlight the superior performance of PEG-ZIF-8-CIP, particularly against younger biofilms, is likely due to its enhanced delivery, stability, and penetration capabilities conferred by PEGylation. This underscores the potential of PEGylated CIP-loaded ZIF-8 (PEG-ZIF-8-CIP) as an effective therapeutic tool for treating biofilm-associated infections, including those caused by resistant strains (Table 2).

**TABLE 2:** MBEC results against *P. aeruginosa* biofilms of 1, 3, and 5 days.

Treatment	Day 1 (mg/mL)	Day 3 (mg/mL)	Day 5 (mg/mL)
PEG-ZIF-8-CIP	0.3	0.42	0.46
ZIF-8-CIP	0.46	0.62	0.64
CIP	0.3	0.6	0.7
ZIF-8	0.72	0.62	0.76

#### 3.4.3 Time-dependent biofilm removal

**FIGURE 8:**
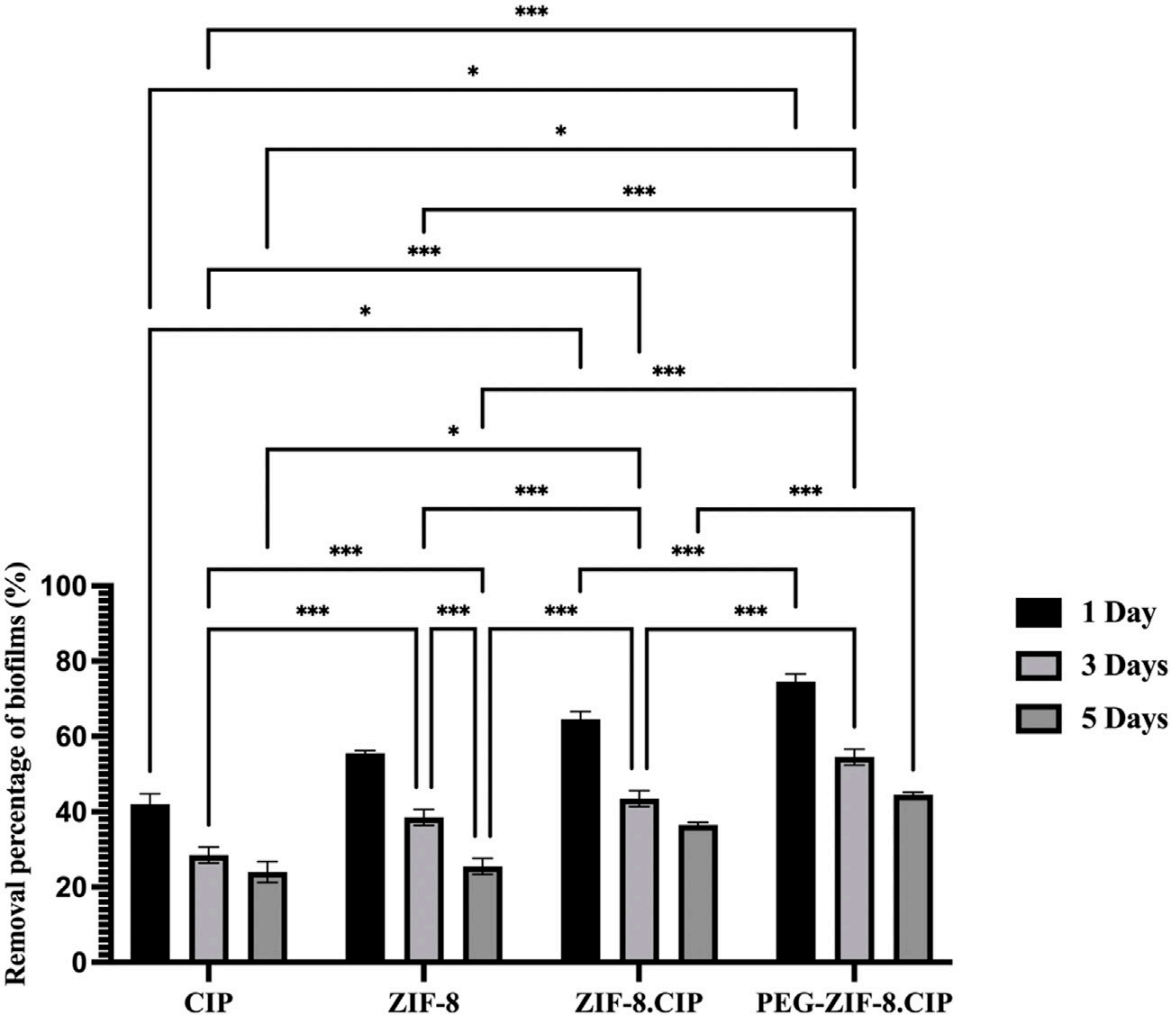
Biofilm removal percentage of *P. aeruginosa* over 1, 3, and 5 days using various treatments. The bar graph illustrates the biofilm removal efficiency of four treatments, consistently exhibiting the highest biofilm removal percentages across all time points. On day 1, PEG-ZIF-8-CIP achieved approximately 74% biofilm removal, while ZIF-8-CIP and ZIF-8 achieved 64% and 55% removal, respectively. The CIP treatment showed the lowest removal efficiency (∼40%). By day 5, PEG-ZIF-8-CIP maintained superior performance, exceeding 44% biofilm removal, while other therapies demonstrated diminished effectiveness. The results highlight the enhanced biofilm removal efficiency of PEG and CIP in synergistic combinations with ZIF-8-based nanozymes. Error bars represent standard deviations, and significant differences between treatments are indicated by asterisks (*p < 0.05, **p < 0.01, ***p < 0.001).

The biofilm removal efficiency of various treatments (CIP, ZIF-8, ZIF-8-CIP, and PEG-ZIF-8-CIP) was evaluated over 1, 3, and 5 days. PEG-ZIF-8-CIP consistently demonstrated the highest biofilm eradication across all time points, highlighting the enhanced therapeutic efficacy provided by PEGylation. As shown in Figure 8, on day 1, PEG-ZIF-8-CIP achieved approximately 74% biofilm removal, significantly outperforming ZIF-8-CIP (64%), ZIF-8 (55%), and CIP alone (42%). By day 3, CIP showed a marked decrease in efficiency, with biofilm removal declining to 28%. ZIF-8 maintained moderate performance at around 38%, while ZIF-8-CIP and PEG-ZIF-8-CIP demonstrated superior removal rates of 43% and 54%, respectively. On day 5, the trend persisted, with CIP displaying limited long-term efficacy at 24% removal. ZIF-8 and ZIF-8-CIP achieved removal rates of approximately 25% and 36% respectively. These results demonstrate that PEGylation enhances the stability and biofilm-targeting capability of CIP-loaded ZIF-8 nanozymes, making PEG-ZIF-8-CIP the most effective treatment against biofilm-associated infections over time.

Tukey’s multiple comparisons test confirmed the differences between treatments and time points. CIP alone showed significant improvement between day 1 and day 3, with a mean difference of 13.500 (p < 0.0001), and between day 1 and day 5, with a mean difference of 18.000 (p < 0.0001). However, CIP’s performance consistently lagged behind ZIF-8-based formulations. Compared to ZIF-8, CIP was significantly less effective on day 1, with a mean difference of −13.500 (p < 0.0001). Similarly, CIP’s performance was outpaced by PEG-ZIF-8-CIP, with a significant difference of −32.500 (p < 0.0001) on day 1 and −20.500 (p < 0.0001) on day 5. ZIF-8 showed moderate antimicrobial activity but was consistently outperformed by PEG-ZIF-8-CIP. On day 1, the difference between ZIF-8 and PEG-ZIF-8-CIP was −19.000 (p < 0.0001). ZIF-8-CIP further enhanced biofilm removal over ZIF-8, with significant differences at all time points, such as -11.000 *(p = 0.0002) on day 5. PEG-ZIF-8-CIP, however, consistently achieved the highest eradication rates, particularly on day 1, with a mean difference of -10.000* (p = 0.0003) compared to ZIF-8-CIP. Comparisons across time points within individual treatments revealed that PEG-ZIF-8-CIP maintained robust biofilm removal efficiency over time. On day 5, it significantly outperformed its activity on day 1 and day 3, with mean differences of 30.000 (p < 0.0001) and 10.000 (p = 0.0003), respectively. These results underscore the superior performance of PEG-ZIF-8-CIP in eradicating biofilms across all tested time points. The statistical significance in most comparisons emphasizes its potential as a robust treatment for biofilm-associated infections.

#### 3.4.4 Cytotoxicity assay

**FIGURE 9:**
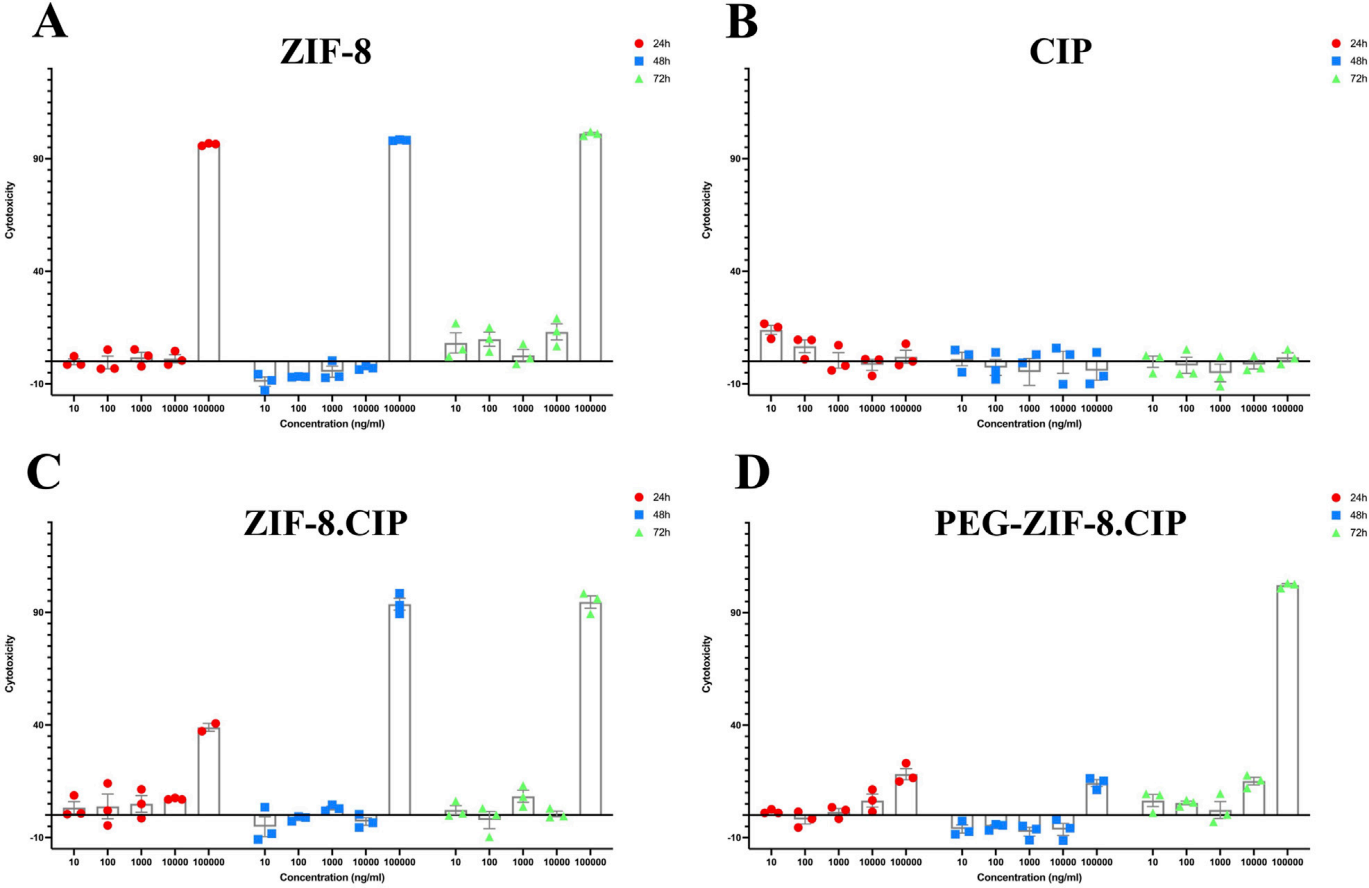
*In vitro* Cytotoxicity of ZIF-8, Free CIP, ZIF-8-CIP, and PEGylated ZIF-8-CIP Nanoparticles in Human Foreskin Fibroblast (HFF) Cells using the MTT Assay. Cells were exposed for 24 h (red circles), 48 h (blue squares), or 72 h (green triangles) to increasing concentrations (10, 100, 1,000, 10,000, and 100,000 ng/mL) of each formulation. Individual symbols represent three independent biological replicates; the mean ± SD is shown by the bars. The horizontal zero line denotes the baseline cytotoxicity of untreated control cells. **(A)** ZIF-8: Low toxicity (≤10% cytotoxicity) at ≤10,000 ng/mL for all time points; at 100,000 ng/mL, viability falls below 10% (i.e., >90% cytotoxicity). **(B)** CIP: Free CIP exhibits minimal toxicity across the whole concentration range, with cytotoxicity remaining below 30% even at 100,000 ng/mL and no appreciable time-dependent effect. **(C)** ZIF-8-CIP: Incorporation of CIP into ZIF 8 modestly decreases toxicity: cytotoxicity is <10% at ≤10,000 ng/mL, ∼40% at 100,000 ng/mL (24 h), and >90% at 100,000 ng/mL (48 and 72 h), with similar profiles at each time point. **(D)** PEG-ZIF-8-CIP: PEGylation significantly reduced cytotoxicity, particularly at 100,000 ng/mL, with the most notable decrease observed at 24 and 48 h.

The cytotoxicity of ZIF-8, ZIF-8-CIP, PEG-ZIF-8-CIP nanozymes, and CIP was evaluated in HFF cells using the MTT assay at 24, 48 and 72 h across concentrations ranging from 10 to 100,000 ng/mL (Figures 9A–D). ZIF-8 showed the highest toxicity at 100,000 ng/mL across all time points. Loading CIP into the ZIF-8 framework initially reduced toxicity; in the ZIF-8-CIP group, cytotoxicity was 38.95% at 24 h but increased sharply to 93.6% at 48 h and 94.6% at 72 h. In contrast, PEG-ZIF-8-CIP exhibited significantly lower cytotoxicity at 24 and 48 h, although no further reduction was observed at 72 h. These results indicate that while ZIF-8 and ZIF-8-CIP nanozymes can be cytotoxic at supratherapeutic doses, PEGylation effectively reduces this toxicity, supporting the potential safety of PEG-ZIF-8-CIP for *in vivo* applications.

### 3.5 In vivo studies

#### 3.5.1 Bacterial load reduction

**FIGURE 10:**
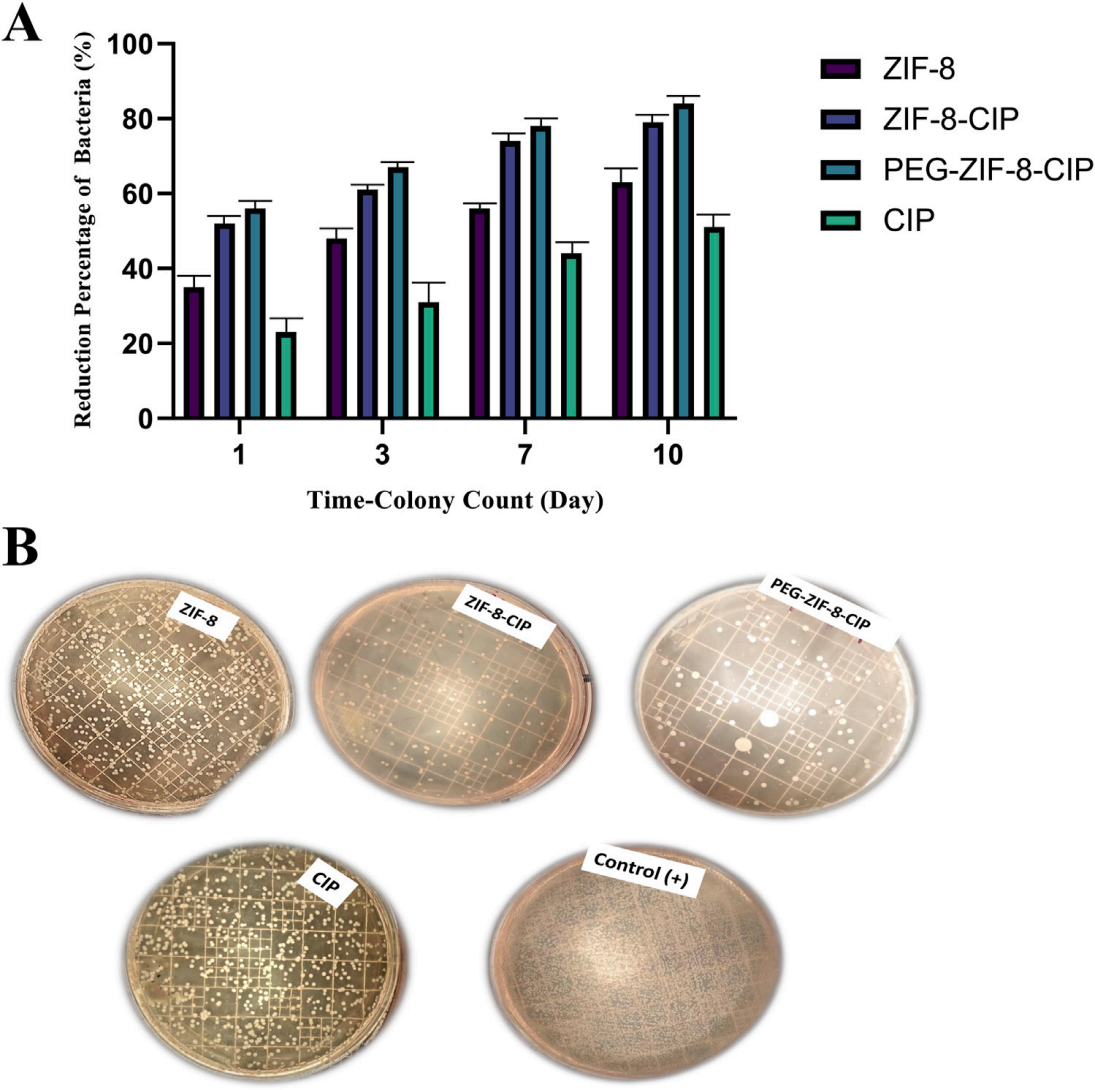
Time-dependent antibacterial efficacy of ZIF-8 formulations against model bacterial strain. **(A)** Quantitative colony reduction over 10 days. Bacterial suspensions were treated with free CIP, bare ZIF-8, ZIF-8-CIP, and PEG-coated ZIF-8-CIP (PEG-ZIF-8-CIP) at equivalent CIP concentrations. Aliquots were plated for colony counts on days 1, 3, 7, and 10 post-treatments. Data (mean ± SD, n = 3) show progressive increases in bacterial killing over time for all formulations. Free CIP achieved a ∼22% reduction by day 1, rising to ∼50% by day 10. ZIF-8 alone produced ∼35% reduction at day 1 and ∼62% by day 10. Incorporation of CIP into ZIF-8 (ZIF-8-CIP) enhanced efficacy, with ∼52% kill on day 1 and ∼79% by day 10. PEG-ZIF-8-CIP showed the highest activity, reaching ∼56% reduction on day 1 and >83% by day 10, indicating synergistic effects of CIP loading and PEGylation on sustained antibacterial action. **(B)** Untreated control (+) displayed dense lawns of colonies. Plates treated with free CIP showed moderate colony suppression, whereas ZIF-8 achieved intermediate reduction. ZIF-8-CIP and PEG-ZIF-8-CIP yielded markedly fewer colonies, with the PEG-coated formulation showing the most pronounced clearance, visually confirming the quantitative data in **(A)**.

Figure 10A shows the time-dependent reduction in wound bacterial burden following *P. aeruginosa* infection and treatment applied 24 h post-wounding. Swab cultures collected on days 1, 3, 7, and 10. As shown in Figure 10B, the group treated with CIP and ZIF-8 (showed the lowest reduction in bacterial percentage, where a significant bacterial presence was observed. In contrast, groups treated with ZIF-8-CIP (and PEG-ZIF-8-CIP demonstrated much higher bacterial reduction at all time points.

ZIF-8 alone trated group produced moderate antibacterial effects, with colony counts declining by 35%, 48%, 56%, and 63% at the respective time points. Incorporation of CIP into the ZIF-8 framework (ZIF-8-CIP) markedly improved clearance, achieving 52%, 61%, 74%, and 79% reductions. PEGylated ZIF-8-CIP further enhanced and sustained bacterial killing, reducing 56%, 67%, 78%, and 84%. In contrast, free CIP displayed the lowest efficacy, yielding only 23%, 31%, 44%, and 51% reductions over the same intervals. These results confirm that drug loading significantly boosts the *in vivo* antibacterial performance of ZIF-8 nanozymes and that PEGylation provides additional benefits in prolonging and amplifying bacterial clearance.

#### 3.5.2 *In vivo* wound healing

**FIGURE 11:**
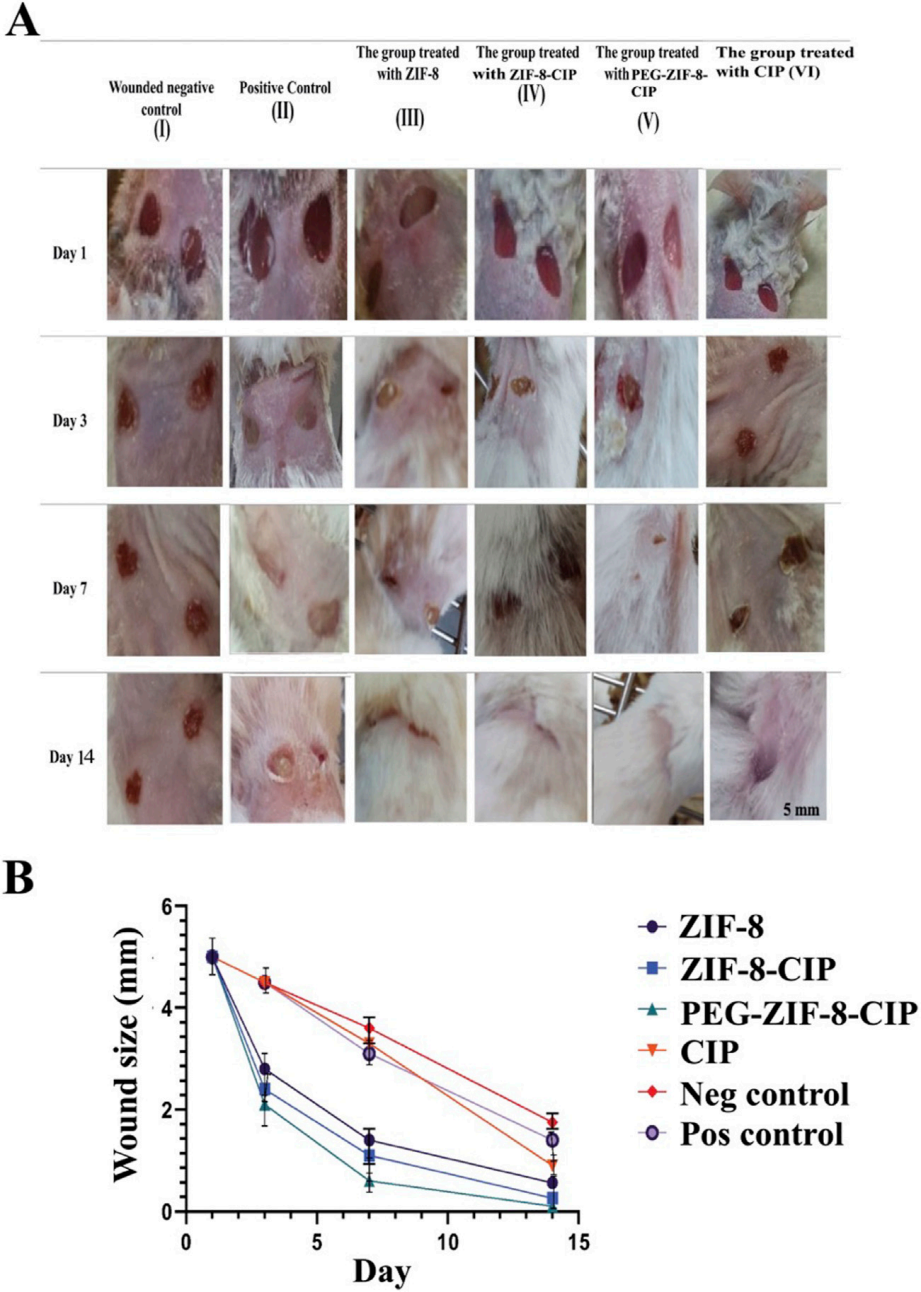
**(A)** Effect of PEG-ZIF-8-CIP on Wounds of Male BALB/c Mice Infected with *P. aeruginosa*. (I) Negative wound control group (without bacterial infection, no treatment). (II) The positive control (wound, bacterial infection, and no treatment). (III) The group was treated with ZIF-8 (wound infected with *P. aeruginosa*). (IV) The group was treated with ZIF-8-CIP (wound infected with *P. aeruginosa*). (V) The group was treated with PEG-ZIF-8-CIP (wound and infected with *P. aeruginosa*). (VI) The group was treated with CIP (wound and infected with *P. aeruginosa*). **(B)** Wound healing rates based on wound sizes were measured over 14 days in different treatment groups of mice. The graph represents the following groups: I (Wounded negative control): Mice without treatment (red line). II (Positive control): Mice treated with standard wound healing agents (purple line). III (ZIF-8): Mice treated with ZIF-8 nanozyme for enhanced wound healing (black line). IV (ZIF-8-CIP): Mice treated with ZIF-8-CIP (blue line). V (PEG-ZIF-8-CIP): Mice treated with PEG-ZIF-8-CIP were applied directly to the wound (green line). VI (CIP): Mice treated with CIP applied directly to the wound (orange line). All groups’ wound sizes decreased over time, with the fastest reduction observed in the PEG-ZIF-8-CIP (V) and the slowest in the Wounded negative control group (I). Time points include measurements on days 1, 3, 7, and 14.

The results showed clear signs of wound healing, including wound closure and hair formation, in the wounds treated with the nanozyme CIP formulation. These wounds healed similarly to non-bacterial contaminated wounds, demonstrating effective treatment on the 14th. In contrast, infected wounds treated with the empty nanozyme or left untreated (positive control) did not show proper wound closure, and hair formation was not observed (Figure 11A).

#### 3.5.3 Wound contraction measurements

Figure 11B illustrates the wound healing progress in male BALB/c mice over 14 days across different treatment groups, including the Wounded Negative Control (I), Positive Control (II), the group treated with ZIF-8 (III), the group treated with ZIF-8-CIP (IV), the group treated with PEG-ZIF-8-CIP (V), and the group treated with CIP (VI): visual observations and comparisons assessed each treatment’s effectiveness in wound healing and epithelialization. The group treated with PEG-ZIF-8-CIP demonstrated the most effective wound healing, showing marked improvement by Day 3 and near-complete recovery by Day 14. In contrast, the Wounded Negative Control group exhibited the slowest healing process, with wounds only partially closing by Day 14. The Positive Control group and those treated with ZIF-8-CIP also showed significant wound contraction, although the healing rate lagged behind PEG-ZIF-8-CIP. Groups treated with ZIF-8 and CIP alone displayed moderate healing, with visible scarring and incomplete recovery by the end of the observation period. The Wounded Negative Control group (Group I) exhibited natural healing with visible reductions in wound size over time. On Day 1, the wounds measured 5 mm and appeared fresh and unaltered. By Day 3, slight reductions in size to 4.5 mm were observed. By Day 7, moderate healing was evident, with wound sizes reducing to 3.6 mm, although the wounds remained visibly open. By Day 14, the wounds had decreased significantly to 1.65 mm but had not completely closed, with scarring present. The Positive Control group (Group II) demonstrated a faster healing trajectory than the negative control. On Day 1, the wounds measured 5 mm and appeared similar to the negative control. By Day 3, the wound size decreased to 4.5 mm, showing reduced inflammation. By Day 7, significant wound contraction was observed, with sizes reducing to 3.1 mm. By Day 14, the wounds were nearly closed, measuring 1.4 mm, with minimal scarring. The group treated with ZIF-8 (Group III) showed better healing outcomes than both control groups. On Day 1, the wounds measured 5 mm and appeared fresh. By Day 3, noticeable improvements were evident, with sizes reducing to 3.4 mm. By Day 7, the wounds exhibited significant healing, decreasing to 1.4 mm. By Day 14, they were nearly closed, measuring 0.7 mm, with minimal visible scars. The group treated with ZIF-8-CIP (Group IV) showed remarkable healing progress. On Day 1, the wounds measured 5 mm. By Day 3, the wounds had reduced significantly to 2.4 mm. By Day 7, near-complete wound closure was observed, with sizes reducing to 1.1 mm. By Day 14, the wounds were almost fully healed, measuring just 0.26 mm, with very faint or no scarring visible. The group treated with PEG-ZIF-8-CIP (Group V) demonstrated the most effective healing among all groups. Starting at 5 mm on Day 1, the wounds contracted significantly by Day 3, measuring 2.1 mm, with reduced redness and inflammation. By Day 7, the wounds were almost completely healed, measuring just 0.7 mm, and by Day 14, the wounds were entirely closed, measuring only 0.1 mm, leaving no visible scars. This highlights the superior efficacy of PEG-ZIF-8-CIP in promoting wound healing and regeneration. The group treated with CIP alone (Group VI) also showed significant healing, though not as effective as PEG-ZIF-8-CIP. The wounds measured 5 mm on Day 1 and reduced to 4.5 mm by Day 3. By Day 7, the size had decreased further to 3.3 mm, and by Day 14, the wounds were nearly healed, measuring 0.9 mm, showing effective healing but slightly slower than the ZIF-8-CIP and PEG-ZIF-8-CIP groups.

#### 3.5.4 Histopathological evaluation

**FIGURE 12:**
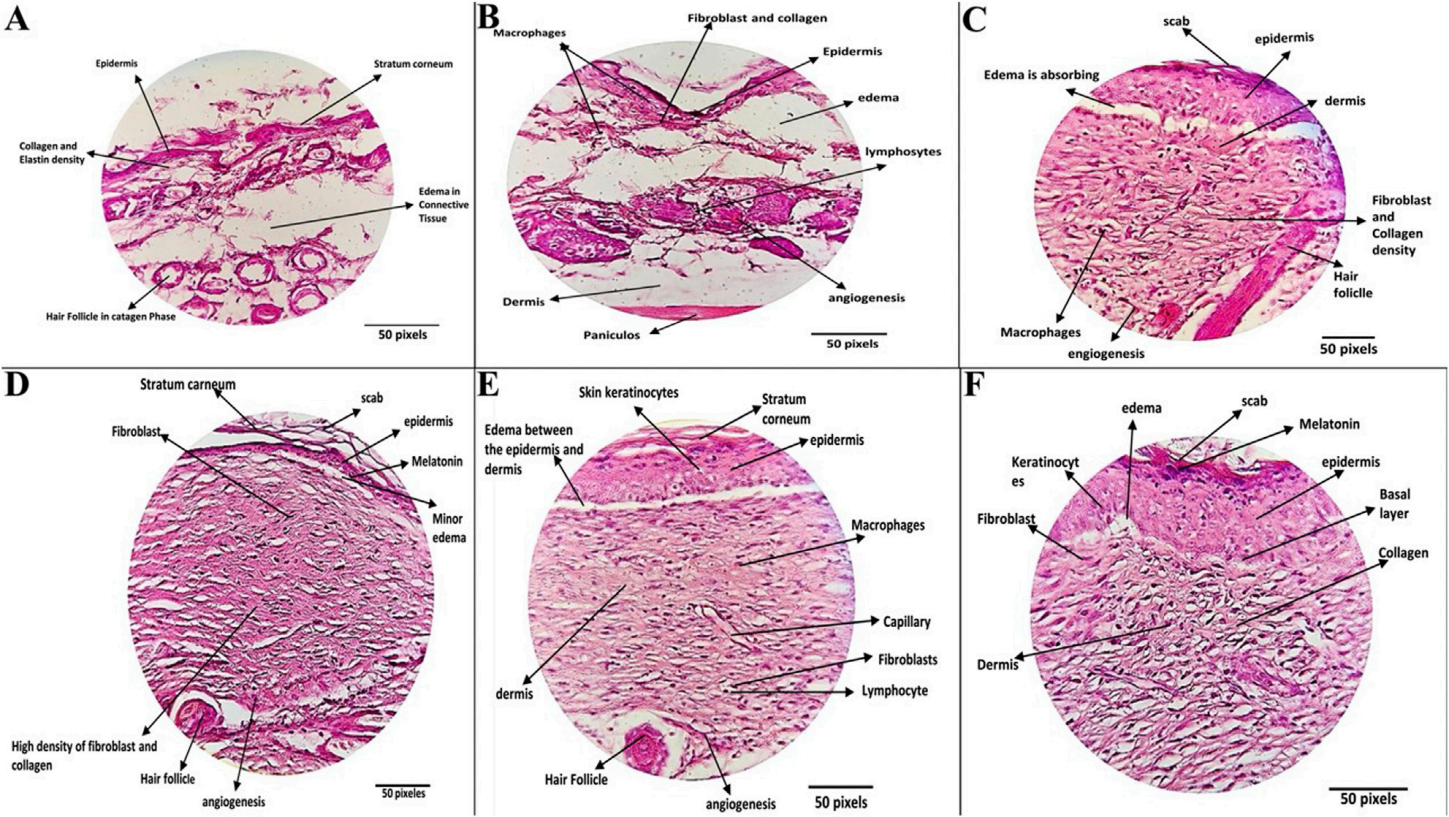
Histopathological Evaluation of Wound Healing Across Different Treatment Groups. **(A)** The Negative Control Group **(**wounded mice without bacterial infection and untreated) showed mild healing characteristics. Wound tissue showed complete restoration, with a fully regenerated epidermis, moderate collagen density, and minimal lymphocytic infiltration in the dermis. Hair follicles were observed in both the anagen and catagen phases, reflecting effective natural healing. **(B)** Positive Control Group: (Wounds mice, wounds infected with *P. aeruginosa* and untreated), exhibiting impaired healing with persistent inflammation and reduced tissue regeneration. The wounds exhibited thinner, more edematous epidermis, increased fibrin deposition, and reduced hair follicle density. Active fibroblasts and macrophages were present, and moderate angiogenesis and persistent inflammation indicated incomplete healing. **(C)** ZIF-8 Treatment Group: (wounded mice, the wounds were infected with *P. aeruginosa* and were treated with a ZIF-8 nanozyme without CIP), demonstrating partial healing with improved dermal and epidermal structures. Wounds treated with ZIF-8 nanozymes demonstrated a thicker epidermis, dense collagen structure, and improved dermal regeneration. Enhanced angiogenesis and organized collagen deposition contributed to effective but partial wound healing. **(D)** ZIF-8-CIP Nanozyme Treatment: (wounded mice, the wounds were infected with *P. aeruginosa* and Wounds treated with CIP-loaded ZIF-8 nanozymes), showing significant regeneration and near-complete repair with minimal inflammation.) Tissue sections displayed nearly normal epidermal thickness, a reticular dermis with dense collagen and elastin deposition, and sparse hair follicles. Highly active fibroblasts, increased angiogenesis, and enhanced melanin pigmentation were observed. Minimal scarring and near-complete wound repair were evident. **(E)** PEG-ZIF-8-CIP Treatment: (wounded mice, the wounds were infected with *P. aeruginosa* and tissue sections from wounds treated with polyethylene glycol-coated ZIF-8-CIP nanozymes), showing optimal healing outcomes with fully restored epidermal and dermal structures and minimal scarring.) The most effective treatment group exhibited complete epidermal regeneration, uniform collagen distribution, and well-formed hair follicles. Minimal edema, enhanced angiogenesis, and normalized dermal and hypodermal structures resulted in significant wound healing and tissue restoration to near-healthy levels. **(F)** CIP Treatment Group: The group was treated with a CIP only (wounded mice, the wounds were infected with *P. aeruginosa*, and the Tissue section was treated with CIP only), demonstrating partial healing with improved dermal and epidermal structures).

Histopathological evaluation of wound tissue samples collected on day 14 from various experimental groups: Wounded Negative Control (I), Positive Control (II), ZIF-8 Nanozyme Treatment (III), and ZIF-8-CIP Treatment (IV), PEG-ZIF-8-CIP Treatment (V), and CIP Treated Group (VI) revealed diverse tissue response patterns, emphasizing the promising therapeutic potential of nanozyme-based treatment (Table 3). Various tissue parameters were assessed using a four-point grading system, including hair follicle reformation, angiogenesis, macrophage presence, neutrophil infiltration, fibroblast activity, collagen deposition, elastin content, inflammation, tissue regeneration, and wound healing. The Wounded Negative Control (I) exhibited high fibroblast activity (score: 5) and collagen deposition (score: 5), along with robust angiogenesis (score: 4). However, hair follicle reformation was minimal (score: 1) and elastin content was low (score: 2). Inflammation and tissue regeneration were moderate (scores: 3 each). In contrast, the Positive Control (II) group with *P. aeruginosa* infection showed high hair follicle reformation (score: 4) and angiogenesis (score: 5) but also significant neutrophil infiltration and inflammation (both score: 3). Elevated fibroblast activity and collagen deposition (both score: 4) were present, alongside substantial fibrosis (score: 5), indicating impaired healing. The ZIF-8 Nanozyme Treatment (III) improved healing compared to the positive control, with moderate hair follicle reformation (score: 3) and robust angiogenesis (score: 4). Neutrophil infiltration remained relatively high (score: 4), while fibroblast activity (score: 3) and collagen deposition (score: 4) were enhanced. Elastin content was low (score: 2), and overall inflammation was moderate (score: 3). Tissue regeneration and wound healing were favorable (both score: 4). Significant advancements were observed in the ZIF-8-CIP Treatment (IV) group, achieving top scores in hair follicle reformation and angiogenesis (both score: 5). Macrophage presence was elevated (score: 4), and neutrophil infiltration was reduced (score: 2), indicating better inflammation control. Fibroblast activity and collagen deposition were balanced (both score: 3), elastin content was moderate (score: 3), and overall inflammation was controlled (score: 3). Tissue regeneration and wound healing were optimal (scores: 4 and 5, respectively). The PEG-ZIF-8-CIP Treatment (V) group demonstrated the most superior healing outcomes, with complete hair follicle reformation and excellent angiogenesis (both score: 5). Macrophage presence remained high (score: 4), and neutrophil infiltration was significantly reduced (score: 4). Fibroblast activity and collagen deposition were strong (both score: 4), while elastin content was minimal (score: 1). Inflammation was excellently controlled (score: 5), tissue regeneration was high (score: 4), and overall wound healing closely resembled healthy tissue (score: 3), indicating comprehensive tissue restoration. Lastly, the CIP Treated Group (VI) showed significant healing with high hair follicle reformation (score: 5), moderate angiogenesis and macrophage presence (both score: 3), and controlled neutrophil infiltration and inflammation (both score: 3). Fibroblast activity and collagen deposition were moderate (both score: 3), elastin content was satisfactory (score: 3), and tissue regeneration was balanced (score: 3). Overall wound healing was effective (score: 3), though not as comprehensive as the PEG-ZIF-8-CIP group. Histopathological analysis of the wounds (after 14 days) revealed that bacterial load in the wound depths was markedly reduced and nearly undetectable in the groups treated with ZIF-8, ZIF-8-CIP, and PEG-ZIF-8-CIP, indicating the strong antibacterial efficacy of these nanozymes (Figure 12).

**TABLE 3:** Histopathological assessment of tissue parameters collected from wounds on day 14.

Parameter	Wounded negative control (I)	Positive control (II)	ZIF-8 group (III)	ZIF-8-CIP group (IV)	PEG-ZIF-8-CIP group (V)	CIP treated group (VI)
Hair follicle	5	2	5	3	4	1
Angiogenesis	3	1	5	4	5	4
Macrophage	3	4	4	3	3	3
Neutrophil	3	4	2	4	3	3
Fibroblast	3	2	3	3	4	5
Collagen	3	2	3	4	4	5
Elastin	3	1	3	2	3	2
Inflammation	3	5	3	3	3	3
Tissue regeneration	3	4	4	4	4	3
Wound healing	3	3	5	4	5	4

## 4 Discussion

*P. aeruginosa*, a major opportunistic pathogen, frequently causes burn wound infections due to its robust biofilm formation and antibiotic resistance (Beig et al., 2021; Beig et al., 2020b; Sharma et al., 2023; Haji et al., 2022). These biofilms impede antibiotic penetration and shield bacteria from the immune system, leading to chronic diseases. This underscores the urgent need for innovative therapeutic strategies like nanozyme-based therapies to target biofilm-associated infections and combat antibiotic resistance effectively (Sahoo and Meshram, 2024; Yin et al., 2022) Nanozyme-based therapies have emerged as promising alternatives due to their intrinsic antimicrobial properties and ability to enhance antibiotic efficacy. Nanozymes, nanomaterials with enzyme-like characteristics, produce ROS that disrupt biofilms and kill bacteria, addressing the challenges of chronic infections (Ma et al., 2023; Pablo-Sainz-Ezquerra et al., 2023; Liao et al., 2022).

In this study, we engineered a PEGylated ZIF-8‐CIP (PEG-ZIF-8-CIP) nanozyme to leverage these advantages. The successful synthesis and characterization of PEG-ZIF-8-CIP confirmed by DLS and zeta potential analyses demonstrated improved homogeneity, colloidal stability, and surface charge relative to uncoated ZIF-8-CIP (Bergaoui et al., 2021; Duan et al., 2023; Costa et al., 2023; Nabipour et al., 2017).

The exceptional colloidal stability of PEG-ZIF-8-CIP retaining a strongly negative zeta potential (−61 to −41 mV) over 180 days underscores PEG’s steric and electrostatic stabilization of the ZIF-8 framework. By contrast, unmodified ZIF-8 drifted to near-neutral charge (−1 mV) and free CIP remained ≈0 mV, reflecting rapid aggregation. Our 6-month stability outperforms previously reported MOF carriers: for example, Bergaouiet al. observed PEG-coated ZIF-8 maintain −50 to −35 mV over 90 days, after which aggregation accelerated (Bergaoui et al., 2021). Such long-term electrostatic resilience is critical for ensuring consistent nanoparticle dispersibility, reproducible dosing, and extended shelf life under ambient conditions.

The *in vitro* CIP release from PEG-ZIF-8-CIP exhibited a classic biphasic, stimuli-responsive profile: an initial burst (∼50% release in 6 h, p < 0.05) from surface-adsorbed drug, followed by a sustained release reaching ≈90% cumulative release at 72 h (Figure 6A). Kinetic modeling confirmed Fickian diffusion (Korsmeyer–Peppas, *R* ^2^ = 0.96, n = 0.26) with strong first-order behavior (*R* ^2^ = 0.94), whereas zero-order and Higuchi fits were inadequate. These results align with analogous MOF systems that reported ∼60% CIP release in 8 h and ∼85% by 48 h from ZIF-8–CIP (Duan et al., 2023), while Costa et al. documented ∼67% release over 96 h from ZnO@ZIF-8 (Costa et al., 2023). The PEG shell in our formulation further smooths the release curve by partially occluding pore entrances, balancing minimal systemic leakage at pH 7.4 with on-demand drug liberation under acidic, infection-like conditions. This sustained, triggerable delivery maintains therapeutic concentrations over days, reducing dosing frequency and potentially limiting resistance development.

Together, these data demonstrate that PEG-ZIF-8-CIP combines long-term colloidal integrity with finely tuned, stimuli-responsive antibiotic release features that position it as a robust platform for localized, controlled drug delivery in wound-infection settings.

Additionally, the nanozyme exhibited SOD-like catalytic activity, further contributing to biofilm destabilization and bacterial killing (Chen et al., 2022; Le Ouay and Stellacci, 2015; Wang R. et al., 2023). This multifunctional design aims to overcome the limitations of conventional antibiotics by combining controlled drug release, ROS generation, and metal‐ion‐mediated antimicrobial effects.

The PEG-ZIF-8-CIP nanoformulation exhibited potent antibacterial activity, significantly outperforming free CIP and the non-PEGylated MOF carrier *in vitro*. This enhanced efficacy can be attributed to a synergistic dual mechanism: the controlled antibiotic release coupled with the inherent antimicrobial effects of the ZIF-8 framework. In our experiments, the PEG-ZIF-8-CIP nanozyme achieved greater killing of *P. aeruginosa* at the desired doses than the drug CIP, indicating nano-synergy. This observation aligns with previous studies on MOF-based antibiotic delivery. For instance, Nabipour et al. encapsulated CIP in nanoscale ZIF-8 and reported heightened inhibition of *Staphylococcus aureus* and *P. aeruginosa* compared to CIP or ZIF-8 alone (Nabipour et al., 2017). Similarly, a recent composite system of ZnO nanoparticles embedded in ZIF-8 (ZnO@ZIF-8) demonstrated that loading CIP into the framework lowered the MIC by order of magnitude or more: the CIP–ZnO@ZIF-8 formulation achieved MIC values up to 10-fold lower against *S. aureus* and ∼200-fold lower against *P. aeruginosa* compared to the MOF without drug, underscoring the remarkable synergy between released antibiotic and the Zn-based nanocarrier (Costa et al., 2023).

The mechanism behind this improved antibacterial efficacy is multifactorial. CIP released from the degrading ZIF-8 primarily targets bacterial DNA gyrase and topoisomerase IV, inducing lethal DNA damage. In parallel, the ZIF-8 framework supplies Zn^2+^ ions upon acid-triggered degradation, and these ions can directly disrupt bacterial physiology. Excess Zn^2+^ is known to exert toxic effects on microbes by interfering with essential enzymes and nutrient metal uptake pathways (for example, competitively inhibiting manganese-dependent processes and disrupting metabolic function) (Chen et al., 2022). Zn^2+^ can also contribute to oxidative stress in bacteria by inhibiting *E*.coli and *S.aureus* antioxidant defenses, effectively amplifying ROS damage in those cells (Chen et al., 2022). Thus, the bactericidal action of the PEG-ZIF-8-CIP system likely stems from a combination of CIP’s precise antibiotic effect and a more nonspecific “metal stress” effect from Zn^2+^, yielding a broad-spectrum activity. This dual action is advantageous against both Gram-positive and Gram-negative pathogens; indeed, a similar MOF-based dual-delivery (Zn^2+^ plus antibiotic) approach using a tetracycline@ZIF-8 composite achieved over 98% bacterial clearance in a difficult infection model, highlighting how MOF-derived metal ions can synergistically boost antibiotic performance (Wang R. et al., 2023).

Compared to other nanotechnologies, our MOF nanoformulation offers comparable or superior antibacterial outcomes while providing controlled release. Silver nanoparticles (AgNPs), for example, are well-established antimicrobial agents known to kill bacteria by releasing Ag ions that disrupt bacterial membranes, denature proteins and generate ROS inside cells (Le Ouay and Stellacci, 2015). AgNPs have broad-spectrum efficacy and have even been combined with antibiotics to achieve synergistic effects (silver can form complexes with antibiotics to enhance their uptake or prevent resistance) (Zheng et al., 2018). Zinc oxide nanoparticles (ZnO NPs) represent another class of nano-antibacterials; ZnO NPs can adhere to bacterial cell walls and, especially under UV or visible light, producing ROS (such as peroxide and superoxide) that damage bacterial membranes and DNA while also slowly releasing Zn^2+^ ions (Sirelkhatim et al., 2015). However, when used alone, both AgNPs and ZnO NPs lack a mechanism to concentrate a specific antibiotic at the infection site. They may require continuous exposure or higher doses to maintain their effects.

In contrast, PEG-ZIF-8-CIP acts as a targeted delivery system: it remains relatively inert in physiological conditions yet releases its antibiotic payload in response to the acidic and enzymatic infection environment. This on-demand release ensures high local concentrations of CIP exactly where needed, likely contributing to the rapid bacterial eradication observed. In summary, the antibacterial efficacy results demonstrate that by integrating a conventional antibiotic into a smart MOF nanocarrier, we achieve an efficacy that matches or exceeds that of traditional nanoparticle antimicrobials while also benefiting from controlled, triggerable drug release and the added antimicrobial actions of the carrier itself.

Beyond planktonic bacteria, our formulation showed a pronounced ability to disrupt and eradicate bacterial biofilms. This is a crucial finding, as biofilm-associated infections are notoriously difficult to treat–biofilter bacteria can withstand antibiotic concentrations hundreds to a thousand times higher than those that kill free-floating bacteria (Ceri et al., 1999). These results indicate that the nanoformulation not only delivers the drug into the biofilm more effectively but may also actively participate in breaking down the biofilm structure.

The mechanisms for biofilm disruption by PEG-ZIF-8-CIP are likely both chemical and physical, as the ZIF-8 particles infiltrate the biofilm and trigger the release of CIP *in situ*. The sustained local release of CIP ensures that even bacteria in the deeper layers of the biofilm are exposed to the antibiotic over an extended period, helping to eliminate persister cells that transiently survive initial antibiotic exposure.

Moreover, ROS generation could be a contributing pathway for biofilm disruption. While ZIF-8 is not a classic ROS generator, zinc ions can indirectly lead to oxidative stress in bacteria by impairing their antioxidant enzymes (Wang X. et al., 2023). Additionally, any uncoordinated ZnO present or formed transiently could catalyze ROS production. It is known that ZnO nanoparticles can produce hydrogen peroxide in aqueous environments and underlie an oxidative attack on biofilms (Sirelkhatim et al., 2015). Indeed, one study demonstrated that nano ZnO treatment inhibited the biofilm formation of *Proteus mirabilis* via ROS-mediated cell damage and EPS degradation (Rajivgandhi et al., 2018). It is plausible that the PEG-ZIF-8-CIP formulation induces similar oxidative stress within the biofilm. The combined action of antibiotic killing and ROS stress effectively breaks the biofilm’s protectivity and eradicates bacterial cells that would otherwise be shielded from antibiotics.

Several points emerge when comparing our approach to other nanotechnologies for biofilm control. AgNPs, for example, have shown anti-biofilm efficacy: silver ions can penetrate biofilms and bind to thiol and amino groups in matrix components and enzymes, compromising biofilms’ structural and metabolic function (Le Ouay and Stellacci, 2015). AgNPs have even been engineered to carry antibiotics or other cargos; by forming complexes with antibiotics, silver nanocarriers can specifically target and eliminate biofilm-embedded bacteria that are resistant to the antibiotic alone (Zheng et al., 2018). These strategies underscore the importance of multi-pronged attacks on biofilms. Our PEGylated MOF system inherently provides a multi-pronged attack (drug + metal ion), akin to a combination therapy delivered in one package. We did not incorporate additional biofilm-matrix degrading enzymes or dispersal molecules in this formulation, but the results suggest that even without those, PEG-ZIF-8-CIP effectively penetrates and collapses biofilm communities. The nanometer-scale size and PEGylation likely aid this penetration, as the PEG corona provides a hydrophilic shell that can diffuse through the polysaccharide-rich EPS and prevents immediate opsonization or sequestration of the particles before they reach the biofilm.

Overall, the biofilm disruption capability of PEG-ZIF-8-CIP is a significant advantage for treating chronic or device-related infections. The formulation addresses a major limitation of conventional antibiotics by clearing biofilm-embedded bacteria. It’s worth noting that many chronic wound infections involve biofilms; hence, this ability is tied to the wound-healing context of our study. The data suggest that our nanoformulation could be leveraged to prevent biofilm establishment on wound dressings or implants or to disperse existing biofilms in infected wounds, thereby restoring the susceptibility of bacteria to antibiotic treatment and immune clearance. This broad antibiofilm performance compares favorably with other advanced approaches and highlights the benefit of integrating MOF-based carriers in anti-infective therapy.

An essential consideration for any biomedical nanoformulation is its biocompatibility. In our MTT assay on HFF, PEG-ZIF-8-CIP nanozymes were well tolerated at antibacterial concentrations, with cell viabilities remaining above 90% after 24 and 48 h. By contrast, uncoated ZIF-8 exhibited pronounced, dose-dependent cytotoxicity across all time points at 100 μg/mL, and ZIF-8-CIP while initially less toxic (38.9% cytotoxicity at 24 h) saw viability plummet to 6.4% by 48 h and 5.4% by 72 h. PEGylation markedly attenuated these effects: the hydrophilic PEG corona stabilizes the framework, reduces protein adsorption and membrane interaction, and slows Zn^2+^ release, in line with previous reports that PEG-modified ZIF-8 shows lower toxicity than its bare counterpart (Xia et al., 2022).

Notably, PEG-coating does not render the formulation completely inert at supratherapeutic doses or extended exposures. At the highest nanoparticle concentrations and after 48–72 h, we observed a measurable decline in cell viability which parallels findings in other MOF systems (e.g., Rutin-loaded ZIF-8 still induced some cytotoxicity at very high doses) (Galandáková et al., 2016). This residual toxicity likely arises from gradual Zn^2+^ buildup and sustained CIP release once the protective PEG shell is overwhelmed, underscoring the importance of dose optimization and controlled release design.

When benchmarked against common antimicrobial nanomaterials, PEG-ZIF-8-CIP offers clear advantages. Silver-based dressings, although broadly effective, impair fibroblast proliferation and keratinocyte migration at concentrations above ∼20–25 μg/mL (Nagoba et al., 2013; Lin et al., 2017), often delaying wound closure. In contrast, our zinc-based MOF achieves similar bacterial killing at lower metal loads leveraging antibiotic delivery rather than heavy-metal toxicity alone and releases Zn^2+^gradually, avoiding the high ionic peaks that trigger oxidative stress in human cells. Zinc-oxide nanoparticles also pose oxidative risks under certain conditions, whereas the slow, frame-driven release from ZIF-8 appears better tolerated.

Taken together, these data demonstrate that PEG-ZIF-8-CIP combines potent antibacterial activity with a favorable safety profile, outperforming both uncoated ZIF-8 and many metal-based antimicrobials when used within its therapeutic window. Its high therapeutic index and controlled release characteristics make it a promising candidate for further *in vivo* evaluation in wound-healing and other localized delivery applications.

Colony counts from wound swabs showed that all ZIF-8–based formulations outperformed free CIP in reducing *P. aeruginosa* burden by day 1, reductions were roughly 35% for ZIF-8, 52% for ZIF-8-CIP, 56% for PEG-ZIF-8-CIP, and only 23% for free CIP; by days 3–10, these climbed into the mid-80% range for PEG-ZIF-8-CIP (67%, 78%, 84%), around 60%–80% for ZIF-8-CIP (61%, 74%, 79%), and 48%–63% for unmodified ZIF-8 (48%, 56%, 63%), while free CIP never exceeded 51%. This stepwise improvement underscores how encapsulating CIP within the ZIF-8 framework boosts local antibiotic concentration, and how the PEG corona extends nanoparticle residence and stability at the wound site. By combining MOF-mediated controlled release with PEG-driven retention, PEG-ZIF-8-CIP offers a robust *in vivo* antimicrobial platform that overcomes the rapid decline in efficacy seen with free drug and leverages both framework and surface chemistry to maximize bacterial clearance.

The histological analysis provided additional insight into the healing quality. Wound sections from the PEG-ZIF-8-CIP group showed thick, well-organized granulation tissue rich in collagen fibers and new capillaries (signs of robust angiogenesis and tissue regeneration). The epidermal layer was reformed, with keratinocytes migrating to cover the wound bed. These observations highlight that rapid bacterial eradication by the PEG-ZIF-8-CIP system directly translates to faster progression through the healing stages (from inflammation to proliferation to remodeling). By removing the infection barrier, nanoformulation allows the body’s natural healing machinery to proceed unimpeded. Beyond infection control, the components of our nano formulation may actively promote wound healing. Zinc is a well-documented beneficial element in wound repair: it plays a role in enzyme systems for DNA synthesis and cell proliferation (Lin et al., 2017).

Our *in vivo* results align with this, as the PEG-ZIF-8-CIP treated wounds not only healed faster but also appeared to have better tissue quality, with more organized collagen bundles and a thicker epidermis, suggesting that zinc’s presence had a positive effect on tissue regeneration. CIP does not directly aid healing (beyond reducing infection), but by curbing the disease, it indirectly prevents the prolonged inflammatory damage that impairs healing. Additionally, the local anti-inflammatory action of Zn^2+^ (discussed in Biocompatibility) could help modulate the wound environment toward regeneration. This creates a more favorable environment for fibroblasts to lay down new matrix and for keratinocytes to re-cover the wound.

Comparatively, conventional treatments for infected wounds, such as silver-based creams, while effective at reducing microbes, often do not show such facilitation of healing and can even retard the process. Silver dressings have been noted to sometimes slow down re-epithelialization due to toxicity to keratinocytes at the wound edge (Lin et al., 2017). In our nanoformulation, no such adverse effect was reported; the re-epithelialization was enhanced. This underscores a key advantage of using a zinc-based MOF with an antibiotic: it is gentle on healing tissues and harsh on bacteria. Other advanced wound care approaches, such as dressings loaded with growth factors or scaffolds releasing zinc or other ions, have attempted to boost healing. Our approach inherently combined antimicrobial and pro-healing elements. For example, ZnO nanoparticles have been incorporated into wound dressings for their antimicrobial and wound-healing properties, exploiting ZnO’s ability to release Zn^2+^ to stimulate cell proliferation (Gonçalves et al., 2017). However, using ZnO alone may not eliminate a heavy infection. In contrast, PEG-ZIF-8-CIP delivered a potent antibiotic to wipe out the infection and concurrently provided zinc to support healing effectively, a one-two punch for infected wounds.

Notably, no significant systemic toxicity or tissue (skin) damage was observed in mice treated with PEG-ZIF-8-CIP. Histopathological analysis revealed no abnormalities attributable to treatment, indicating that the effects of the formulation were localized to the wound area without off-target adverse effects. This is likely due to the controlled release (limiting systemic CIP exposure) and the fact that any nanoparticles that enter the circulation are PEGylated and, therefore, less susceptible to opsonization and accidental tissue deposition. Over time, the MOF degrades to zinc ions and biodegradable ligands that the body can handle; the zinc can be absorbed or excreted, and the 2-methylimidazole is metabolized or eliminated. The lack of minimal toxicity is an encouraging sign of the biocompatibility of the formulation in whole organism tissue, complementing the *in vitro* cellular safety data.

In summary, the *in vivo* wound healing performance of PEG-ZIF-8-CIP was excellent. The formulation cleared the infection more effectively than standard antibiotic treatment and actively accelerated the healing process, improving healing outcomes. These results position PEG-ZIF-8-CIP as a promising therapeutic for infected wounds, where antimicrobial action and tissue repair support are needed. Integrating antimicrobial MOFs in a wound dressing or injectable form could significantly reduce healing times and improve recovery in patients with chronic or severe wound infections. Our study provides a proof-of-concept for such an integrated approach. With further optimization and validation (including clinical trials), it could be translated into a novel nano-enabled wound therapy that addresses the key challenges of infection control and tissue regeneration.

While substantial, our panel of 60 CIP-resistant *P. aeruginosa* isolates may not capture burn-wound pathogens’ full genomic and phenotypic diversity. Future work should expand to larger, geographically and epidemiologically diverse isolate collections and include additional clinically relevant species to confirm the broad applicability of our nanozyme’s antimicrobial and anti-biofilm performance.

One limitation of our work is that biofilm disruption was evaluated solely in static, mono-species *in vitro* assays, which cannot fully recapitulate the multifactorial environment of wound biofilms *in vivo* where host tissues, immune effectors, fluid shear forces, nutrient gradients, and polymicrobial interactions all modulate matrix architecture and antimicrobial penetration. Although these assays offer critical proof-of-concept for PEG-ZIF-8-CIP’s intrinsic anti-biofilm activity, they may not predict performance in complex clinical settings. Future studies must therefore challenge our nanozyme under more physiologically relevant conditions—using dynamic flow-cell reactors to simulate shear stress, *ex vivo* human skin explants to include host extracellular matrix and cell populations, and animal wound-infection models to capture immune responses and interspecies biofilm dynamics to validate its efficacy against true wound biofilms.

Another limitation of our work is the exclusive reliance on a murine burn-wound model to assess PEG-ZIF-8-CIP’s efficacy. Rodent skin differs from human skin in epidermal thickness, collagen organization, immune-cell composition, and intrinsic healing rates, which can influence nanoparticle penetration and tissue-repair kinetics. Consequently, the promising antimicrobial and pro-healing results observed in mice may not fully predict human outcomes. Future studies need to validate these findings in a porcine burn model—whose skin architecture and wound-healing dynamics more closely resemble those of humans—and ultimately in early-phase clinical trials. This stepwise approach mirrors the development pathways of other nanozyme-based wound therapies and will help ensure that PEG-ZIF-8-CIP’s human performance aligns with our preclinical data (Mo et al., 2022).

Despite the promising performance of PEG-ZIF-8-CIP in accelerating wound closure and eradicating infection, several limitations must be acknowledged. While accelerated healing was observed, the precise molecular mechanisms underlying wound repair and how these nanozymes influence signaling pathways such as ROS-mediated modulation of growth factor expression, zinc-dependent enzyme activation, and immune cell recruitment were not explored in detail. Moreover, the interplay between ROS generation, Zn^2+^ signaling, and host immune responses remains to be elucidated. Long-term safety beyond the 14-day observation period and the potential for immune sensitization or chronic inflammation induced by repeated nanozyme exposure also warrant thorough investigation. Finally, translating this formulation toward clinical use will require overcoming manufacturing challenges particularly achieving consistent PEGylation and reproducible CIP loading under GMP conditions and validating scalability. Future work should, therefore, integrate detailed mechanistic studies of both biofilm disruption and tissue-regenerative signaling, extended preclinical safety and immunogenicity assessments over longer durations, optimization of nanoparticle processing for large-scale production, and ultimately controlled clinical trials in burn-wound patients to confirm efficacy, safety, and mechanism.

## 5 Conclusion

PEGylated ZIF-8‐CIP (PEG-ZIF-8-CIP) nanozyme offers a novel and effective strategy for managing biofilm-associated infections caused by multidrug-resistant *P. aeruginosa*. Our formulation achieves potent planktonic killing and superior biofilm disruption by combining controlled CIP release with ROS-generating activity within a stable, PEG-coated metal-organic framework. *In vitro* and *in vivo* evaluations demonstrated its dual functionality: effectively eradicating biofilms and simultaneously promoting wound healing, as evidenced by accelerated re-epithelialization, robust granulation tissue formation, and minimal scarring.

These results establish a strong foundation for developing next-generation nanozyme-based therapies to address the growing challenges of antibiotic resistance and chronic wound management. The on-demand release mechanism ensures high local concentrations of CIP at infection sites—enhancing antimicrobial efficacy while minimizing systemic exposure and toxicity. Excellent biocompatibility and a lack of significant inflammatory responses further underscore the clinical readiness of this approach.

Taken together, these insights highlight the transformative potential of PEG-ZIF-8-CIP to revolutionize the treatment of biofilm-related infections and advance care for bacterial infections. Although our findings are highly promising, large-scale clinical trials and long-term safety evaluations will be essential to confirm broader feasibility and optimize its use across diverse patient populations.

## Data Availability

The raw data supporting the conclusions of this article will be made available by the authors, without undue reservation.

## Supplementary material

SUPPLEMENTARY FIGURE S1 Molecular Detection of the *oprL* Gene and CIP Resistance Profiling in *Pseudomonas aeruginosa* Isolates. **(A)** Agarose gel electrophoresis of PCR products amplified with *oprL*-specific primers. Lanes 1–6 and 7–12 represent clinical isolates; M, 100-bp DNA ladder; +, positive control (*P. aeruginosa* ATCC 27853); –, negative control. A clear 504-bp band confirms the *oprL* gene in all tested isolates. **(B)** Broth microdilution assay in a 96-well plate for CIP susceptibility in *Pseudomonas aeruginosa* isolates. Rows A (1–12) to G (1–12): individual isolates. Wells 1–11 (A–G): serial dilutions of CIP from 512 μg/mL to 0.25 μg/mL. Well 12 (A12–H12): positive control (known CIP-resistant strain). Row H (H1–H11): negative controls (media only). Turbidity indicates bacterial growth; MIC values (the lowest concentration showing no turbidity) were ≥ 2 μg/mL for all isolates, confirming CIP resistance.
